# Collision cross sections of large positive fullerene molecular ions and their use as ion mobility calibrants in trapped ion mobility mass spectrometry

**DOI:** 10.1007/s00216-024-05579-0

**Published:** 2024-10-09

**Authors:** Tobias Oppenländer, Jürgen H. Gross

**Affiliations:** Institute of Organic Chemistry, Heidelberg University, Im Neuenheimer Feld 270, 69120 Heidelberg, Germany

**Keywords:** Laser desorption/ionization (LDI), Fullerene soot, Trapped ion mobility spectrometry (TIMS), Ion mobility calibrant, Matrix-assisted laser desorption/ionization (MALDI), Electrospray ionization (ESI)

## Abstract

Positive-ion laser desorption/ionization (LDI) of fullerenes contained in soot as produced by the Krätschmer-Huffman process delivers a wide range of fullerene molecular ions from C_56_^+•^ to above C_300_^+•^. Here, the collision cross section (CCS) values of those fullerene molecular ions are determined using a trapped ion mobility-quadrupole-time-of-flight (TIMS-Q-TOF) instrument. While CCS values in the range from C_60_^+•^ to C_96_^+•^ are already known with high accuracy, those of ions from C_98_^+•^ onward had yet to be determined. The fullerene molecular ions covered in this work have CCS values from about 200 to 440 Å^2^. The fullerene molecular ion series is evenly spaced at C_2_ differences in composition, and thus, small CCS differences of just 2.2–3.5 Å^2^ were determined across the entire range. Fullerene M^+•^ ions may be employed as mobility calibrants, in particular, when very narrow 1/*K*_0_ ranges are being analyzed to achieve high TIMS resolving power. In addition, due to the simple elemental composition, M^+•^ ions of fullerenes could also serve for mass calibration. This study describes the determination of CCS values of fullerene molecular ions from C_56_^+•^ to C_240_^+•^ and the application of ions from C_56_^+•^ to C_220_^+•^ to calibrate the ion mobility scale of a Bruker timsTOFflex instrument in any combination of LDI, matrix-assisted laser desorption/ionization (MALDI), and electrospray ionization (ESI) modes in the CCS range from about 200 to 420 Å^2^. This use was exemplified along with ions from Agilent Tune Mix, leucine-enkephalin, angiotensin I, angiotensin II, and substance P.

**Graphical Abstract:**

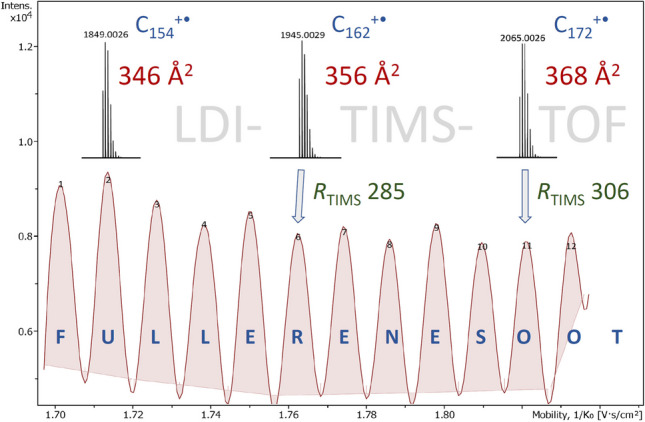

**Supplementary Information:**

The online version contains supplementary material available at 10.1007/s00216-024-05579-0.

## Introduction

During the last two decades, ion mobility mass spectrometry coupling has become a widespread analytical tool [1–6] that has been commercialized in various technical implementations [6–9]. Among these technical variants, the instrument used here relies on trapped ion mobility spectrometry (TIMS). Introduced in 2011 [10], TIMS has gained importance, and thus, the development and the general properties of TIMS are discussed in a number of publications [11–18].

Fullerene soots as produced by the famous Krätschmer-Huffman process cover a wide range of pure carbon molecules [19, 20], and moreover, have become commercially available. The typical composition of such a fullerene soot has early on been analyzed by secondary ion mass spectrometry, and somewhat dependent on the solvent used for extraction, fullerenes up to C_420_ were detected [21]. Very recently, fullerene soot extract was “re-discovered” as a potential resource for a large variety of fullertubes [22].

A few years ago, fullerene soot was extensively studied by negative-ion electrospray ionization (ESI) and positive-ion atmospheric pressure chemical ionization (APCI) using a trapped ion mobility-quadrupole-time-of-flight (TIMS-Q-TOF) instrument and collision cross section (CCS) values of a wide range of fullerene ions were determined with great accuracy [23]. This study provided the CCS values of singly charged fullerene anions from C_60_^–•^ to C_150_^–•^, of doubly charged fullerene anions from C_70_^2–^ to C_140_^2–^, and of singly charged positive fullerene ions from C_60_^+•^ to C_96_^+•^. However, M^+•^ ions of fullerenes larger than those of C_96_ were apparently not obtained by APCI. While this study using APCI and TIMS delivered CCS values of 210.0 Å^2^ for C_60_^+•^ and 226.6 Å^2^ for C_70_^+•^ [23], earlier work using drift tube ion mobility separation yielded slightly larger values of 213.1 Å^2^ for C_60_ ions and 231.4 Å^2^ for C_70_ ions [24] which might be due to a contribution of [M + H]^+^ ions, because there, ESI was used to form positive fullerene ions.

Positive-ion laser desorption/ionization (LDI) does generate M^+•^ ions of large fullerenes with ease and typically yields ions from C_60_^+•^, *m/z* 720, to at least C_180_^+•^, *m/z* 2160, when fullerene soot is analyzed this way. Operating the LDI source at high laser flux may even allow desorption/ionization to generate ions like C_240_^+•^, *m/z* 2880, and beyond.

When a Bruker timsTOFflex instrument with an ESI-to-MALDI switchable ion source is employed, either ionization technique can be used without physically changing the instrument configuration. This particular instrument can thus be mass-calibrated in ESI mode for use in LDI or matrix-assisted laser desorption/ionization (MALDI) and vice versa. Furthermore, it may be calibrated for ion mobility analyses in either mode. The established procedure of ion mobility calibration based on the Agilent ESI Tune Mix presents the best standard for CCS calibration across a wide range available to date [25]. Nonetheless, Agilent Tune Mix has some limitations when narrow ranges of ion mobilities are to be studied. In the case of TIMS, the scale is inversed ion mobility, 1/*K*_0_ [Vs cm^–2^], and on this scale, the reference ions are at Δ(1/*K*_0_) of about 0.16–0.26 [25]. Additionally, with an ESI/MALDI combination ion source, CCS calibration in ESI mode can lead to a decrease in CCS accuracy when the interface temperature in ESI mode accidentally differs from that in LDI or MALDI modes. The reason for changes in ion mobility arises as interface temperature, in particular settings of desolvation gas temperature and flow, affects the pressure inside the TIMS funnel, and thus, causes slight shifts in 1/*K*_0_. Therefore, it is desirable to have an easy-to-use TIMS calibration in LDI or MALDI modes that can also be used across very narrow 1/*K*_0_ ranges. Very recently, an approach of using bis-MPA dendrimers for CCS calibration provided calibrants usable in both ESI and MALDI from as low as 160 Å^2^ up to a very high limit of 1700 Å^2^, however, also spaced at wider gaps [26].

Provided a proper CCS standard has been used for calibration, the TIMS device permits the determination of accurate CCS values of unknown compounds across a wide range [11, 18, 25–29]. The only prerequisite for correct CCS values is that the calibration has been performed at exactly the same pressure and 1/*K*_0_ range that is to be used for the subsequent analysis of the unknown. Having a proper and easy-to-use CCS standard providing narrow gaps between the reference ions could thus be beneficial when high TIMS resolving power is required, e.g., to separate isomers.

Moreover, having a standard for LDI would enable TIMS separations and CCS measurements in LDI and MALDI without the need to switch to ESI as required when using the Agilent Tune Mix. Vice versa, it would allow for such experiments in ESI mode without interruptions due to the necessity of admitting Tune Mix because a quick switch to LDI allows for TIMS calibration. A recent study on dendrimer standards provided exactly these capabilities for wide ranges and up to very large CCS values [26]. Admittedly, these advantages are only available when an instrument with an ESI-to-MALDI switchable ion source is being used.

The aim of this work is to (i) expand the range of CCS values to fullerene molecular ions from C_98_^+•^ to C_240_^+•^ and (ii) describe their application as convenient and narrowly spaced CCS calibrant across the range of 200–420 Å^2^.

## Experimental

A trapped ion mobility-quadrupole-time-of-flight (TIMS-Q-TOF) instrument (timsTOFflex, Bruker Daltonics, Bremen, Germany) was used. The instrument was equipped with an ESI-to-MALDI switchable combination ion source (Bruker DualSource MTP). The mass spectrometer was controlled by the Bruker timsControl software (V 5.1) and data analysis was performed using the Bruker DataAnalysis software (V 6.1).

To ensure constant pressure in the TIMS device even when switching between modes, the interface was operated using nitrogen desolvation gas at 4.0 l min^–1^ and 200 °C for ESI, LDI, and MALDI modes alike.

Initially, external mass calibrations were established in ESI mode by using Agilent Tune Mix (G1969-85,000) for the *m/z* 300–3000 range [30, 31]. Mass accuracy was generally in the order of 2 ppm. The initial ion mobility calibration was performed using the same Tune Mix [25]. Later, the CCS values of fullerene molecular ions were also employed as TIMS calibrants.

ESI-TIMS spectra were obtained by accumulation of 15 s of measurement time, while LDI-TIMS and MALDI-TIMS spectra were generated by summing 10 packages of 100 laser shots each, i.e., 10 ms accumulation per package and 1000 laser shots per final spectrum.

Fullerene soot as produced by the Krätschmer-Huffman synthesis was obtained from Sigma-Aldrich. The soot was suspended in toluene by sonication and 1 µl of this suspension was applied per spot on the stainless steel sample plate and allowed to dry.

## Results and discussion

### Range of fullerene ions and some basic considerations

**Fig. 1:**
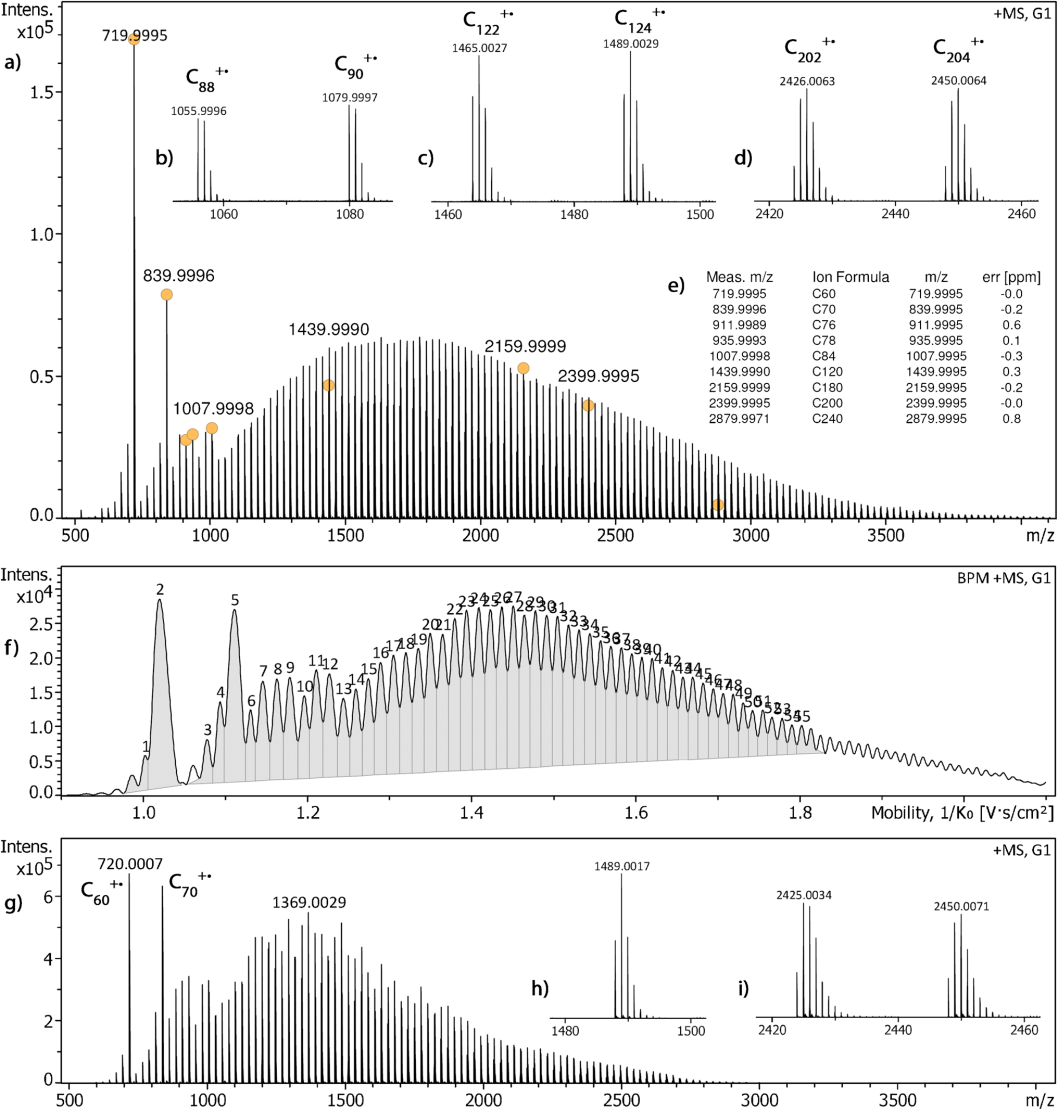
Positive-ion LDI spectra of fullerene soot extract as obtained under different conditions to illustrate the effect of TIMS. (**a**) LDI spectrum as obtained while TIMS had been turned off, where insets (**b**) to (**d**) show expanded views of signals from different *m/z* ranges. In (**e**), a list of formulas based on accurate mass highlights some of the fullerenes along the series while dots at the peaks in (**a**) indicate the ions in this list. Part (**f**) shows the base peak mobilogram (BPC, 1/*K*_0_ range 0.90–2.10, ramp time 500 ms, accumulation 10 ms, 2.30 mbar) with 55 compounds assigned. Finally, (**g**) to (**i**) present the corresponding LDI-TIMS sum spectrum covering a markedly reduced *m/z* range and show insets with isotopic patterns from the higher *m/z* section

In the positive-ion LDI spectrum, fullerene molecular ions are observed across a wide range, where the ions detected range from C_50_^+•^ to about C_300_^+•^, i.e., they reach notably above *m/z* 3500, as long as TIMS is turned off (Fig. 1). The insets in Fig. 1 show expanded views of signals from different *m/z* ranges and demonstrate excessive resolution at any point of the spectrum. A list of selected formulas determined by accurate mass identifies some of the fullerenes along the series. Further, there are no other ion series between the fullerene ions of even carbon number, neither doubly charged ions of larger fullerenes, nor fragments of odd carbon number, nor fullerene oxides. Interferences from ions other than C_n_^+•^ (*n* = 56, 58, 60, …, 240) could therefore be ruled out.

In the context of the present study, some characteristics of the TIMS device should briefly be reconsidered as a few present limitations while others offer advantages. First of all, with a suitable CCS standard at hand, the TIMS device offers the determination of accurate CCS values of unknown compounds across any range covered by that standard [11, 18, 25–29].

When using TIMS, the range of ion mobilities that can be transmitted under the same operational conditions is limited by the 1/*K*_0_ range that can simultaneously be handled by the TIMS funnel. This causes a cutoff at either end of the range, and thus, from the wide distribution of fullerene ions displayed in Fig. 1. As the pressure in the TIMS funnel may intentionally be set in the 1.7–3.0 mbar range, the section of 1/*K*_0_ values, i.e., the range of CCS values, that can be handled under given conditions can be adjusted to the actual needs. The ranges exhibited in Fig. 1f and g are as wide as we were able to achieve in one run in terms of 1/*K*_0_ and *m/z* range, respectively.

**Fig. 2:**
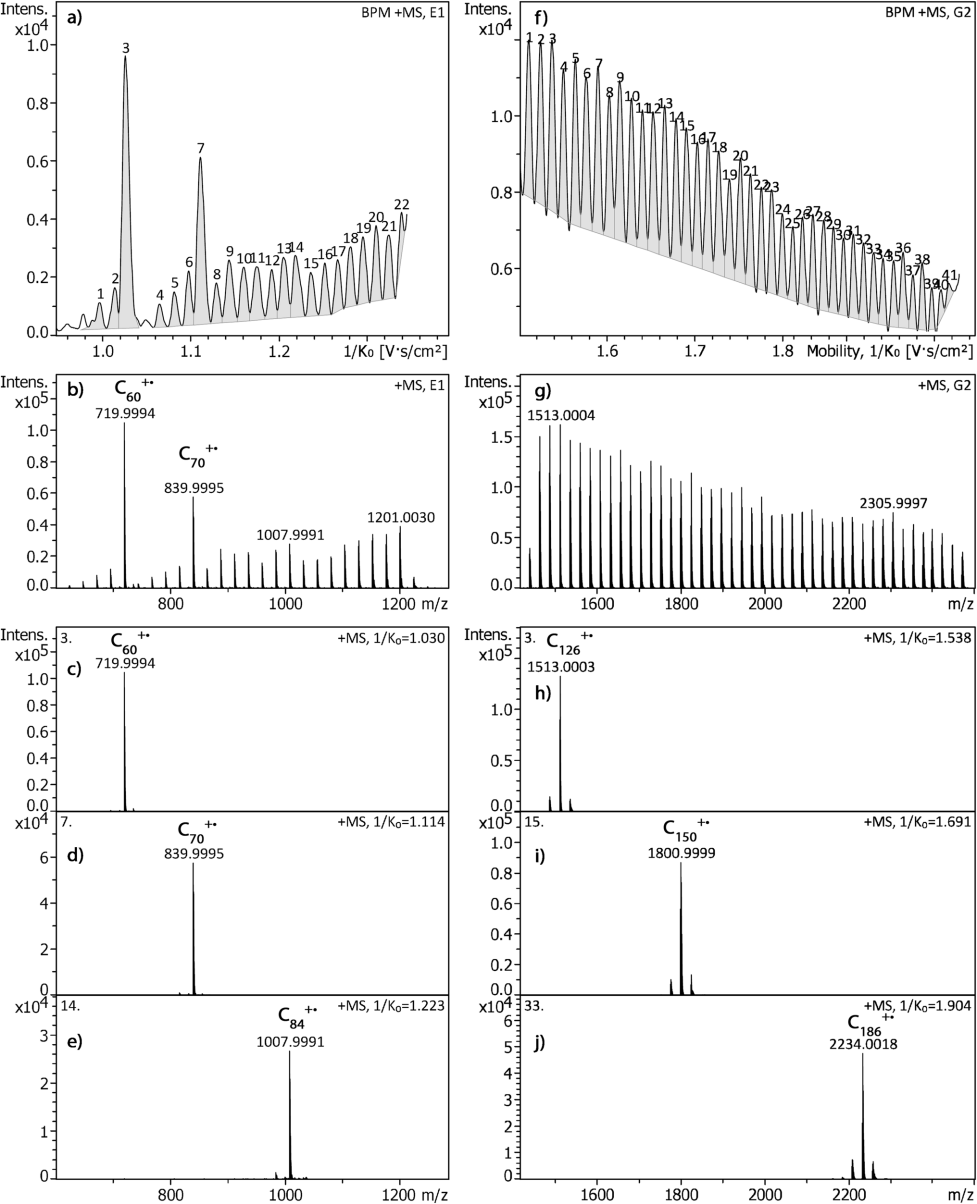
Positive-ion LDI-TIMS spectra of fullerene soot extract under different TIMS conditions illustrating the TIMS ranges and the effect of TIMS settings on resolving power: (**a**) 1/*K*_0_ 0.95–1.35, ramp 500 ms, accum. 10 ms, 2.75 mbar versus (**f**) 1/*K*_0_ range 1.60–2.00, ramp 500 ms, accum. 10 ms, 2.45 mbar. Parts (**b**) and (**g**) show the corresponding sum spectra covering C_54_^+•^ to C_100_^+•^ and C_142_^+•^ to C_202_^+•^, respectively. Finally, (**c**) to (**e**) and (**h**) to (**j**) show examples of fullerene molecular ion peaks assigned to compounds as separated by TIMS. The numbers in the upper left corner of spectra are the compound numbers of the mobilogram peaks

Also typical of TIMS, wide 1/*K*_0_ ranges, short ramp times, and lower pressure result in reduced ion mobility resolution. Vice versa, low ion population in the TIMS funnel, narrow 1/*K*_0_ ranges combined with long ramp times, preferably at higher pressure, fosters higher resolving power of TIMS [11, 15, 32]. In MALDI, the low ion population is commonly achieved by accumulation of a low number of laser shots per ion package sent into the TIMS device [33]. This interplay of operational parameters is illustrated by combining a comparatively long ramp time of 500 ms with narrow 1/*K*_0_ ranges (Fig. 2). The upper limit of CCS is also defined by the maximum electric field available to hold back ions within the TIMS funnel. It can be expanded by lowering the pressure because of a reduced aerodynamic force on the ions at lower pressure (Fig. 2). Thus, ions having a large CCS become accessible when the TIMS is operated at reduced pressure, e.g., at 2.0–2.3 mbar.

While the TIMS resolution (*R*_TIMS_) in the analysis covering the wide 1/*K*_0_ range shown in Fig. 1f was at medium level (*R*_TIMS_ ≈ 70 for C_70_^+•^ and *R*_TIMS_ ≈ 110 for C_120_^+•^), the narrow lower range displayed in Fig. 2a delivered much better resolution (*R*_TIMS_ ≈ 120 for C_70_^+•^ and *R*_TIMS_ ≈ 170 for C_96_^+•^) and the narrow higher range performed even better (*R*_TIMS_ ≈ 220 for C_124_^+•^ and *R*_TIMS_ ≈ 340 for C_196_^+•^, Fig. 2f).

### Low level of fragmentation

Fullerene molecular ions such as C_60_^+•^ and C_70_^+•^ are known to undergo stepwise fragmentation by losses of multiple C_2_ units [34, 35]. Thus, one might argue that the prominent peaks at *m/z* 672 and 696 displayed in the mass spectra of Figs. 1 and 2 are due to fragmentation of C_60_^+•^ ions. Fragmentation of the ions of the standard would be disadvantageous as superimposition of different related species might reduce the accuracy of the CCS measurement via peak widening and multiple charge states within one spectrum could cause similar problems, too [28, 29, 36]. We have therefore analyzed a sample of [60]fullerene at different levels of the laser fluence. While the molecular ion at *m/z* 720, C_60_^+•^, remained by far the base peak, the relative intensities of some easier-to-ionize impurities increased as the laser fluence was reduced (Supplementary Material Fig. S1). At lower laser fluence, these antioxidant impurities as well as C_60_O^+•^, *m/z* 736, were ionized more efficiently than the fullerene due to the heteroatoms present in these molecules, thereby causing their relative intensities to rise. In contrast, the peak due to the C_58_^+•^ ion, *m/z* 696, decreased in intensity along with a drop of the absolute intensity of the C_60_^+•^ ion. As laser-induced fragmentation appeared to be irrelevant for observed amount of C_56_^+•^ and C_58_^+•^ ions, we also acquired tandem mass spectra of C_60_^+•^ ions using collision-induced dissociation (CID) at different collision energies. In these CID spectra, no fragment ions were observed at collision offset voltages up to 150 V (Supplementary Material Fig. S2). Finally, the C_58_^+•^ fragment ion, *m/z* 696, appeared when the laser fluence was notably increased in addition. Even then, the relative intensity of the C_58_^+•^ fragment ion was just 0.12%. Thus, fragmentation of C_60_^+•^ should not be accounted for the occurrence of C_56_^+•^ and C_58_^+•^ ions in the spectra of the fullerene soot extract. The small fullerenes were rather present as components of this extract. Accordingly, one may assume that fragmentation of large fullerenes did also not play a relevant role here and that at least an overwhelming portion of any of the ions appearing in LDI spectra were representing molecular ions of fullerenes of various sizes.

### Determination of collision cross sections

The following measures were applied to achieve reliable collision cross sections of the fullerene molecular ions: (i) Sufficient TIMS resolution was realized by setting narrow 1/*K*_0_ ranges of Δ(1/*K*_0_) = 0.30–0.50 with 0.50 being the most frequently employed interval, (ii) these Δ(1/*K*_0_) ranges generally covered three Tune Mix calibrant ions, (iii) the CCS values were determined in sets of three to four runs each with recalibration of the TIMS device after each or least after every second run, (iv) these sets were repeated on different days with slightly varying Δ(1/*K*_0_) and TIMS pressures, and (v) they were acquired by either of the authors.

To exclude user bias, the component mass spectra were obtained by employing the function “Find Components–Mobilogram” using the default settings provided in DataAnalysis 6.1. For each compound detected, the program then delivered the CCS values. In some cases, when the program would not automatically deliver the CCS values even though the compound had been detected and a corresponding 1/*K*_0_ value was displayed in the compound list, we used the Compass Mobility Calculator to manually fill the gap. The CCS values were transferred to Microsoft Excel spreadsheet application (Office 2019) to determine averages, standard deviations, and deviations from published values as far as these were available. A small portion of this data as obtained from one specific set of measurements is listed in Table 1 to explain the procedure applied here, while the complete data is provided in the Supplementary Material (Table S1).

**Table 1:** Determination of CCS values of positive fullerene molecular ions. The difference between this specific dataset and the reference values from Ref. [23] is calculated when available

			TIMS 0.95–1.45, 500 ms, 10 ms, 2.80 mbar, file Nos						
		CCS [Å^2^]							
Nominal *m/z*	Molecular formula	Ref. [23]	10493	10494	10495	Average	Stand. Dev. [%]	Delta CCS [Å^2^]	Delta CCS [%]
672	C_56_		203.4	203.8	204.0	203.7	0.1		
696	C_58_		206.8	207.2	207.3	207.1	0.1		
720	C_60_	210.0	209.4	209.8	209.9	209.7	0.1	0.3	0.1
744	C_62_								
768	C_64_		217.4	217.4	218.0	217.6	0.1		
792	C_66_		220.9	221.1	221.4	221.1	0.1		
816	C_68_		224.1	224.4	224.7	224.4	0.1		
840	C_70_	226.6	226.8	227.1	227.3	227.1	0.1	− 0.5	− 0.2
864	C_72_		230.6	230.9	231.2	230.9	0.1		
888	C_74_		233.4	233.8	233.9	233.7	0.1		
912	C_76_	236.2	236.4	237.2	237.3	237.0	0.2	− 0.8	− 0.3
936	C_78_	239.2	239.9	240.2	240.4	240.2	0.1	− 1.0	− 0.4
960	C_80_		243.3	243.7	243.8	243.6	0.1		
984	C_82_	245.2	246.3	246.5	246.7	246.5	0.1	− 1.3	− 0.5
1008	C_84_	247.9	249.0	249.3	249.5	249.3	0.1	− 1.4	− 0.5
1032	C_86_	251.1	252.7	252.7	253.1	252.8	0.1	− 1.7	− 0.7
1056	C_88_	254.5	255.8	255.9	256.1	255.9	0.0	− 1.4	− 0.6
1080	C_90_	257.3	259.0	259.1	259.2	259.1	0.0	− 1.8	− 0.7
1104	C_92_	260.1	261.9	261.9	262.2	262.0	0.1	− 1.9	− 0.7
1128	C_94_	263.3	264.9	265.0	265.2	265.0	0.0	− 1.7	− 0.7
1152	C_96_	266.0	268.0	267.9	268.1	268.0	0.0	− 2.0	− 0.7
1176	C_98_		271.0	270.8	271.0	270.9	0.0		
1200	C_100_		273.9	273.8	273.9	273.9	0.0		
1224	C_102_		276.8	276.9	277.0	276.9			
1248	C_104_		279.8	279.7	279.8	279.8			
1272	C_106_		282.6	282.6	282.7	282.6			
1296	C_108_		285.6	285.3	285.5	285.5			
1320	C_110_		288.4	288.2	288.4	288.3			
1344	C_112_		291.3	291.0	291.2	291.2			

For example, the dataset in Table 1 comprised three runs and was obtained after setting a range of 1/*K*_0_ = 0.95–1.45 (ramp time 500 ms, accumulation 10 ms, 2.80 mbar) that covered the ions from C_56_^+•^ to C_112_^+•^. It had thus been calibrated using the Tune Mix ions at nominal *m/z* 622 (1/*K*_0_ = 0.991), 922 (1/*K*_0_ = 1.199), and 1222 (1/*K*_0_ = 1.393). The complete set of compound lists, compound spectra, and a calibration report are compiled in Figs. S3–S6. The values in Table 1 show that the standard deviations of CCS values between single runs were in the order of 0.1%, and more importantly, the values were in very good agreement with the published CCS values [23] as far as these were available within less than 1%.

Analogous datasets were measured by selecting different 1/*K*_0_ ranges mostly using Δ(1/*K*_0_) = 0.50 and by choosing the boundaries as to achieve some overlap to previous and following ranges. The series of CCS values could thus be extended from the established ones to hitherto unknowns up to C_240_^+•^. All of those sets of determinations are compiled in Table S1. While differences in CCS between smaller neighboring fullerene ion like C_56_^+•^ to C_80_^+•^ are in the order of 3.5 Å^2^ and also are subject to higher variation, they were found to markedly decrease to about 2.3 Å^2^ in the range around C_180_^+•^. Such diminishing differences are reasonable in that the growth of fullerenes by C_2_ units presents an increasingly smaller relative change of the entire molecule the larger they get.

Careful examination of TIMS resolution values across the series of ions, e.g., of those covered in Figs. S4–S6, reveals a reduced resolution of the two peaks corresponding to C_60_^+•^ and C_70_^+•^ in comparison to their less abundant neighbors. This can be caused by space charge effects inside the TIMS device due to the high charge density which appears unavoidable when mixtures with so many components are being studied. Notably lower laser power would not have been sufficient to desorb the larger fullerenes, and therefore, was not an option here. Regardless of TIMS resolution, it should be noted that the CCS values always were in the same narrow ranges within say ± 1 Å^2^ independent of which 1/*K*_0_ ranges and laser settings had actually been used as demonstrated by going along lines in Table S1.

Looking at the compound mass spectra in Fig. 2, one might argue that the separation of the large fullerene M^+•^ ions is not sufficient for accurate CCS measurements. While the fullerenes in the 1/*K*_0_ range of 0.95–1.35 are completely separated, the C_n_^+•^ signals corresponding to the 1/*K*_0_ range of 1.60–2.00 are accompanied by about 10% of both C_n–2_^+•^ and C_n+2_^+•^ ions. Nonetheless, the peaks in the mobilogram are highly symmetrical and one may assume that the underestimation caused by the contribution of some C_n–2_^+•^ should be balanced by the slight overestimation due to some C_n+2_^+•^ ions.

The ion population in the TIMS device caused by a large number of fullerene species could also be affecting the CCS values as compared to that obtained when a single compound is being analyzed. This was checked by measuring the CCS value using the sample of C_60_ already dealt with in Figs. S1 and S2, which was not different from values obtained from mixtures.

All CCS values determined during this study across various ranges are compiled in Table S1 while the CCS averages from there were taken as the final results and are listed in Table 2.

**Table 2:** Positive fullerene molecular ions and their CCS values

Molecular ion	CCS [Å^2^]	Molecular ion	CCS [Å^2^]	Molecular ion	CCS [Å^2^]
C_56_^+•^	203.2	C_118_^+•^	299.1	C_180_^+•^	377.7
C_58_^+•^	206.6	C_120_^+•^	301.8	C_182_^+•^	380.1
C_60_^+•^	209.3	C_122_^+•^	304.8	C_184_^+•^	382.3
C_62_^+•^	214.0	C_124_^+•^	307.4	C_186_^+•^	384.6
C_64_^+•^	217.0	C_126_^+•^	310.1	C_188_^+•^	386.9
C_66_^+•^	220.7	C_128_^+•^	312.7	C_190_^+•^	389.2
C_68_^+•^	223.9	C_130_^+•^	315.4	C_192_^+•^	391.4
C_70_^+•^	226.5	C_132_^+•^	317.9	C_194_^+•^	393.6
C_72_^+•^	228.8	C_134_^+•^	320.5	C_196_^+•^	395.9
C_74_^+•^	233.1	C_136_^+•^	323.0	C_198_^+•^	398.1
C_76_^+•^	236.5	C_138_^+•^	326.6	C_200_^+•^	400.2
C_78_^+•^	239.5	C_140_^+•^	329.2	C_202_^+•^	402.4
C_80_^+•^	242.9	C_142_^+•^	331.7	C_204_^+•^	404.6
C_82_^+•^	245.8	C_144_^+•^	334.2	C_206_^+•^	406.9
C_84_^+•^	248.6	C_146_^+•^	336.8	C_208_^+•^	409.1
C_86_^+•^	252.0	C_148_^+•^	339.3	C_210_^+•^	411.2
C_88_^+•^	255.0	C_150_^+•^	341.7	C_212_^+•^	413.3
C_90_^+•^	258.1	C_152_^+•^	344.2	C_214_^+•^	415.5
C_92_^+•^	261.1	C_154_^+•^	346.7	C_216_^+•^	417.7
C_94_^+•^	264.0	C_156_^+•^	349.2	C_218_^+•^	419.8
C_96_^+•^	267.0	C_158_^+•^	351.6	C_220_^+•^	422.0
C_98_^+•^	270.0	C_160_^+•^	354.0	C_222_^+•^	424.6
C_100_^+•^	273.1	C_162_^+•^	356.5	C_224_^+•^	426.3
C_102_^+•^	276.2	C_164_^+•^	358.9	C_226_^+•^	428.6
C_104_^+•^	279.1	C_166_^+•^	361.3	C_228_^+•^	430.6
C_106_^+•^	281.9	C_168_^+•^	363.6	C_230_^+•^	433.0
C_108_^+•^	284.8	C_170_^+•^	366.0	C_232_^+•^	434.4
C_110_^+•^	287.7	C_172_^+•^	368.4	C_234_^+•^	436.6
C_112_^+•^	290.5	C_174_^+•^	370.6	C_236_^+•^	439.2
C_114_^+•^	293.0	C_176_^+•^	373.2	C_238_^+•^	441.3
C_116_^+•^	296.4	C_178_^+•^	375.4	C_240_^+•^	442.9

### Cross-checking of the collision cross sections

Even though the datasets appeared consistent and in accordance with previously published reference values as far as these were available, one would like to obtain some validation of the CCS values of C_98_^+•^ and larger ions. For a cross-check, the fullerene CCS values from Table 2 were thus used to build a reference file. Using this fullerene-based CCS reference file to calibrate the TIMS device, we then determined the CCS values of some known compounds. While the CCS values of fullerene molecular ions up to C_240_^+•^ were determined, we decided to restrict the use of those values as a reference to C_220_^+•^ (422.0 Å^2^) due to limited TIMS resolution in the highest range.

First, the fullerene-based calibration was employed to determine the CCS of ions delivered in ESI mode by Agilent Tune Mix. The procedure was analogous to the employed for the analyses of the fullerenes in terms of 1/*K*_0_ ranges and ramp times. The CCS values of Tune Mix ions were found in very good agreement with the published standard values (Table 3). Admittedly, the determination of CCS values of Tune Mix ions using ions as a reference the CCS of which have been determined based on a Tune Mix calibration bears some characteristic of a circular reasoning.

**Table 3:** CCS values of standard compounds determined based on a mobility calibration using the fullerene molecular ions

Compound	Ionic formula	*m/z* (calc.)	CCS reference [Å^2^]	CCS Exp. [%]	Delta CCS [Å^2^]	Delta CCS [%]
Tune Mix	[C_12_H_19_F_12_N_3_O_6_P_3_]^+^	622.0290	202.96	202.6	− 0.3	− 0.2
Tune Mix	[C_18_H_19_F_24_N_3_O_6_P_3_]^+^	922.0098	243.20	242.7	− 0.5	− 0.2
Tune Mix	[C_24_H_19_F_36_N_3_O_6_P_3_]^+^	1221.9906	282.20	281.3	− 0.9	− 0.3
Tune Mix	[C_30_H_19_F_48_N_3_O_6_P_3_]^+^	1521.9715	316.96	316.8	− 0.1	0.0
Tune Mix	[C_36_H_19_F_60_N_3_O_6_P_3_]^+^	1821.9523	351.25	351.8	0.5	0.1
Tune Mix	[C_42_H_19_F_72_N_3_O_6_P_3_]^+^	2121.9332	383.03	383.7	0.6	0.2
Tune Mix	[C_48_H_19_F_84_N_3_O_6_P_3_]^+^	2421.9140	412.96	413.7	0.7	0.2
Leucine-enkephalin	[C_28_H_38_N_5_O_7_]^+^	556.2766	229.80	226.1	− 3.7	− 1.6
Angiotensin II	[C_50_H_72_N_13_O_12_]^+^	1046.5418	313.87	313.1	− 0.8	− 0.2
Angiotensin I	[C_62_H_90_N_17_O_14_]^+^	1296.6848	356.14	354.4	− 1.7	− 0.5
Substance P	[C_63_H_99_N_18_O_13_S]^+^	1347.7354	360.54	360.5	0.0	0.0

**Fig. 3:**
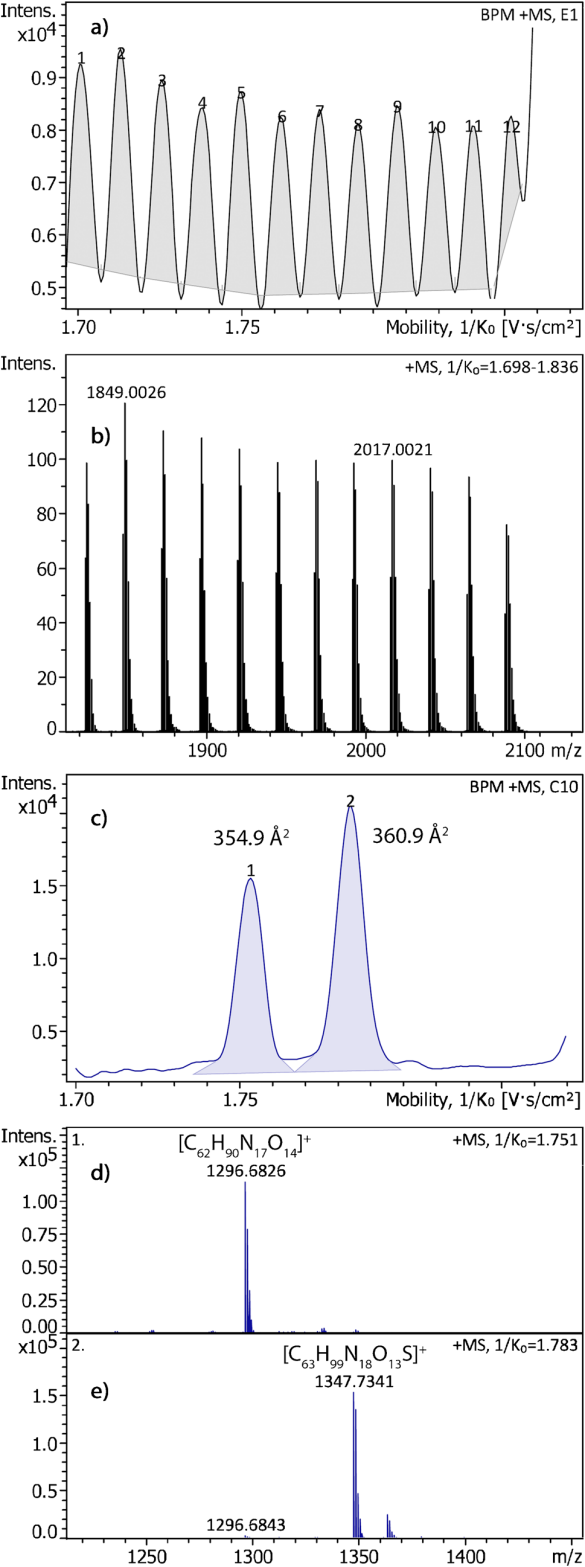
Application of a fullerene TIMS calibration across a narrow 1/*K*_0_ range. (**a**) Mobilogram of the positive-ion LDI-TIMS spectrum of fullerene soot to calibrate the TIMS range 1/*K*_0_ 1.70–1.85 (ramp 500 ms, accum. 10 ms, 2.50 mbar) and (**b**) the corresponding sum spectrum showing all fullerene ions in that range, i.e., from C_152_^+•^ to C_174_^+•^. Next, the [M + H]^+^ ions of angiotensin I and substance P as analyzed by MALDI under the same TIMS conditions resulting in (**c**) two well-separated peaks in the mobilogram ([C_62_H_90_N_17_O_14_]^+^, CCS_exp_ 354.9 Å^2^, and [C_63_H_99_N_18_O_13_S]^+^, CCS_exp_ 360.9 Å.^2^) along with their compound mass spectra (**d**) and (**e**)

Therefore, some small peptides were also analyzed. The CCS values of the [M + H]^+^ ions angiotensin II ([C_50_H_72_N_13_O_12_]^+^, 313.87 Å^2^), angiotensin I ([C_62_H_90_N_17_O_14_]^+^, 356.14 Å^2^), and substance P ([C_63_H_99_N_18_O_13_S]^+^, 360.54 Å^2^) [25] were determined by using fullerenes in LDI mode to calibrate the TIMS device and MALDI mode to create the peptides ions. The application of a fullerene-based TIMS calibration across the 1/*K*_0_ range of 1.70–1.85 (ramp 500 ms, accum. 10 ms, 2.50 mbar) is demonstrated in Fig. 3. The mobilogram of the fullerene ions shows a TIMS resolution of 275 for C_152_^+•^ increasing to 306 for C_174_^+•^. Based on this calibration, the [M + H]^+^ ions of angiotensin I and substance P were analyzed by MALDI under the same TIMS conditions. This delivered two well-separated peaks in the mobilogram ([C_62_H_90_N_17_O_14_]^+^, CCS_exp_ 354.9 Å^2^, and [C_63_H_99_N_18_O_13_S]^+^, CCS_exp_ 360.9 Å^2^). Different ranges were employed and all were found to yield CCS values in good agreement with the published values (Table 3). As these three peptides only covered the upper half of the CSS range of fullerenes, leucine-enkephalin ([C_28_H_38_N_5_O_7_]^+^, 229.8 Å^2^) [37] was also analyzed this way. All datasets leading to the values in Table 3 are provided in the Supplementary Material (Table S2). For even more confidence, the CCS values of the four peptides were also measured using the Tune Mix TIMS calibration and found to be very close to those obtained using the fullerene-based TIMS calibration (Table S2). In particular, the CCS value of leucine-enkephalin (229.8 Å^2^ according to Ref. [37]) was also found at the slightly lower value of 227.1 Å^2^, i.e., closer to the results based on the fullerene calibration which yielded 226.18 Å^2^. Here, the difference of the fullerene-based average value of 226.18 Å^2^ to the published CCS was somewhat larger than in the previous cases and may, at least in part, be attributed to the fact that a different technique, i.e., traveling wave ion mobility spectrometry (TWIMS), had been used there [37].

The accuracy of the CCS values of angiotensin I and II as well as of substance P achieved here supports our previous assumption that some overlap with C_n–2_^+•^ and C_n+2_^+•^ ions in the mobilogram does not shift the CCS values of C_n_^+•^ ions as their contributions are symmetrical on either side of the major compound.

It is noteworthy that the *m/z* range of fullerenes and peptides of similar CCS values differs notably in that the fullerenes have much lower CCS values at the same *m/z*. For example, fullerenes roughly covering the *m/z* 1800–2100 range are needed to calibrate the CCS scale for peptides around *m/z* 1300. This shows how compact the roughly spherical fullerene ions are in comparison to molecules with branched chains like peptides. Finally, the consolidated CCS values determined here are in line with the published experimental and calculated values that were correlated with (essentially) spherically shaped fullerenes [23].

### Practical advice when using fullerenes as reference

Using a set of narrowly spaced reference ions comes with the risk of mis-assigning a signal as the CCS differences of around 3 Å^2^ on average may cause the calibration algorithm to erroneously start the procedure with a neighboring signal leading to similar yet systematically shifted, and thus, incorrect CCS values. This risk was found greatly reduced, when a basic TIMS calibration using Tune Mix was performed in ESI mode before switching to narrow ranges by using the fullerene in LDI mode. The reference file (in Bruker file format) with accurate *m/z* values of monoisotopic fullerene molecular ions from C_56_^+•^ (*m/z* 671.9995, 203.2 Å^2^) to C_220_^+•^ (*m/z* 2639.9995, 422.0 Å^2^) is provided as Supplementary Material.

Another concern could be due to the use of fullerene soot that, especially in the case of larger fullerenes, contains various isomers. Laser desorption/ionization might also cause isomerization, and the isomer composition might even vary from batch to batch of the soot.

However, as already demonstrated in the previous study, the limit of TIMS resolution does just permit a differentiation of the C_78_ isomers [23], whereas one may exclude relevant peak broadening for the larger species. The isomers simply are too similar in CCS, and thus, should not affect the final results in terms of peak position and peak width. Anyway, at any stage of CCS determinations, a cross-check against Tune Mix can be recommended for maintaining best accuracy.

## Conclusions

Herein, we described the determination of CCS values of M^+•^ ions of fullerenes from C_56_^+•^ to C_240_^+•^ by LDI-TIMS-MS, among which the CCS values of those from C_98_^+•^ to C_240_^+•^ had been unknown so far. The CCS values determined here were found to correlate well with those expected for fullerenes of (more) spherical shapes. Then, we employed the ion series from C_56_^+•^ to C_220_^+•^ for ion mobility calibration in order to validate the CCS values by cross-checking them via the determination of CCS values of several standards. Thereby, we also demonstrated the application of those fullerene molecular ions as narrow-spaced TIMS calibrants covering a CCS range of about 200–420 Å^2^ for use in any combination with MALDI and ESI analyses.

## Supplementary Information

Below is the link to the electronic supplementary material.

Supplementary file1 (PDF 2876 KB)

Supplementary file2 (PDF 3.05 KB)
