# Supplementary material for: Collision cross sections of large positive fullerene molecular ions and their use as ion mobility calibrants in trapped ion mobility mass spectrometry
Source: Anal Bioanal Chem. 2024 Oct 9;416(28):6187–97. doi: 10.1007/s00216-024-05579-0 (PMC11541393; doi:10.1007/s00216-024-05579-0)
Supplement: Supplementary file 1 — Supplementary file1 (PDF 2876 KB) [file 216_2024_5579_MOESM1_ESM.pdf]

Supplementary Material

Collision cross sections of large positive fullerene molecular ions and their use as ion mobility calibrants in trapped ion mobility mass spectrometry

Tobias Oppenländer, Jürgen H. Gross

Institute of Organic Chemistry, Heidelberg University, Im Neuenheimer Feld 270,

69120 Heidelberg, Germany.

Send correspondence to Jürgen H. Gross

ORCID 0000-0003-0748-2535

email juergen.gross@oci.uni-heidelberg.de

phone +49/6221/54-8409

Fig. S1. Laser desorption/ionization mass spectra of [60]fullerene at different laser fluences. While the $C_{60}^{+\bullet}$ ion remains by far the base peak at *m/z* 720, the relative intensities of some impurities (sample repeatedly re-dissolved in the same PE vial) increase as the laser fluence is decreased. We assume that the antioxidant impurities at *m/z* 647 and 663 (Irgafos type) as well as $C_{60}O^{+\bullet}$, *m/z* 736, are still well ionized at the lower setting due to the heteroatoms present, thereby causing their relative intensities to rise. In contrast, the peak due to the $C_{58}^{+\bullet}$ ion, *m/z* 696, decreases in intensity as the $C_{60}^{+\bullet}$ ion peak drops in absolute intensity (8×10^5 counts at 40 % to 9×10^4 counts at 20 %). The relative intensities are noted next to the peaks, the formulas of the components are assigned in the spectrum at the bottom.

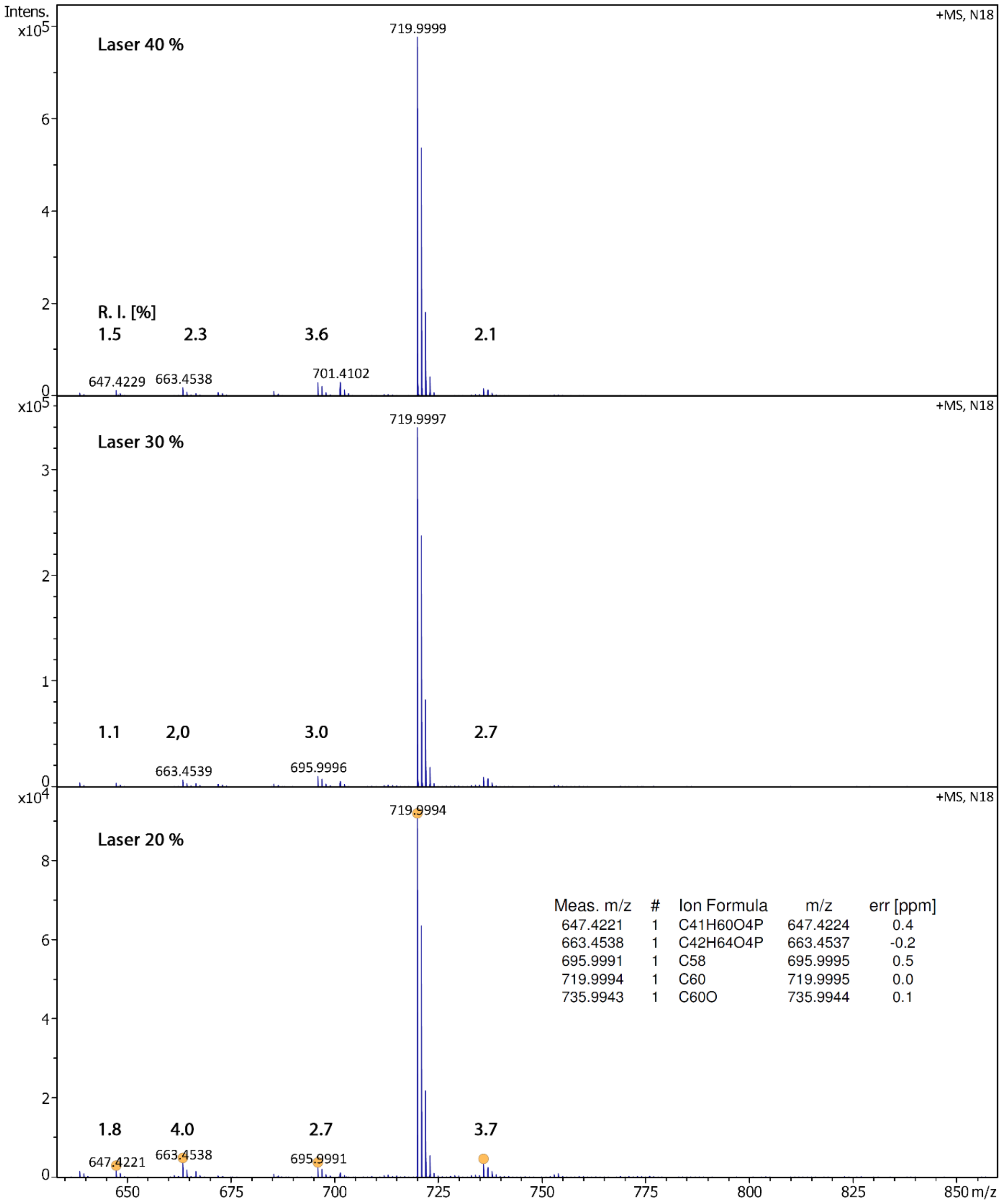

Fig. S2. Tandem mass spectra of $C_{60}^{+\bullet}$ generated by LDI at different collision offset voltages and settings of laser fluence (noted in the spectra). The $C_{60}^{+\bullet}$ ion does not undergo any fragmentation, even at a collision offset of 150V. The $C_{58}^{+\bullet}$ fragment ion, *m/z* 696, only appears when the laser fluence is notably increased in addition. Even then, the relative intensity of the $C_{58}^{+\bullet}$ fragment ion is just 0.12 %. Thus, fragmentation of $C_{60}^{+\bullet}$ should not be the reason for $C_{56}^{+\bullet}$ and $C_{58}^{+\bullet}$ to occur in the spectra of fullerene soot extract. The small fullerenes are rather present as components in this extract.

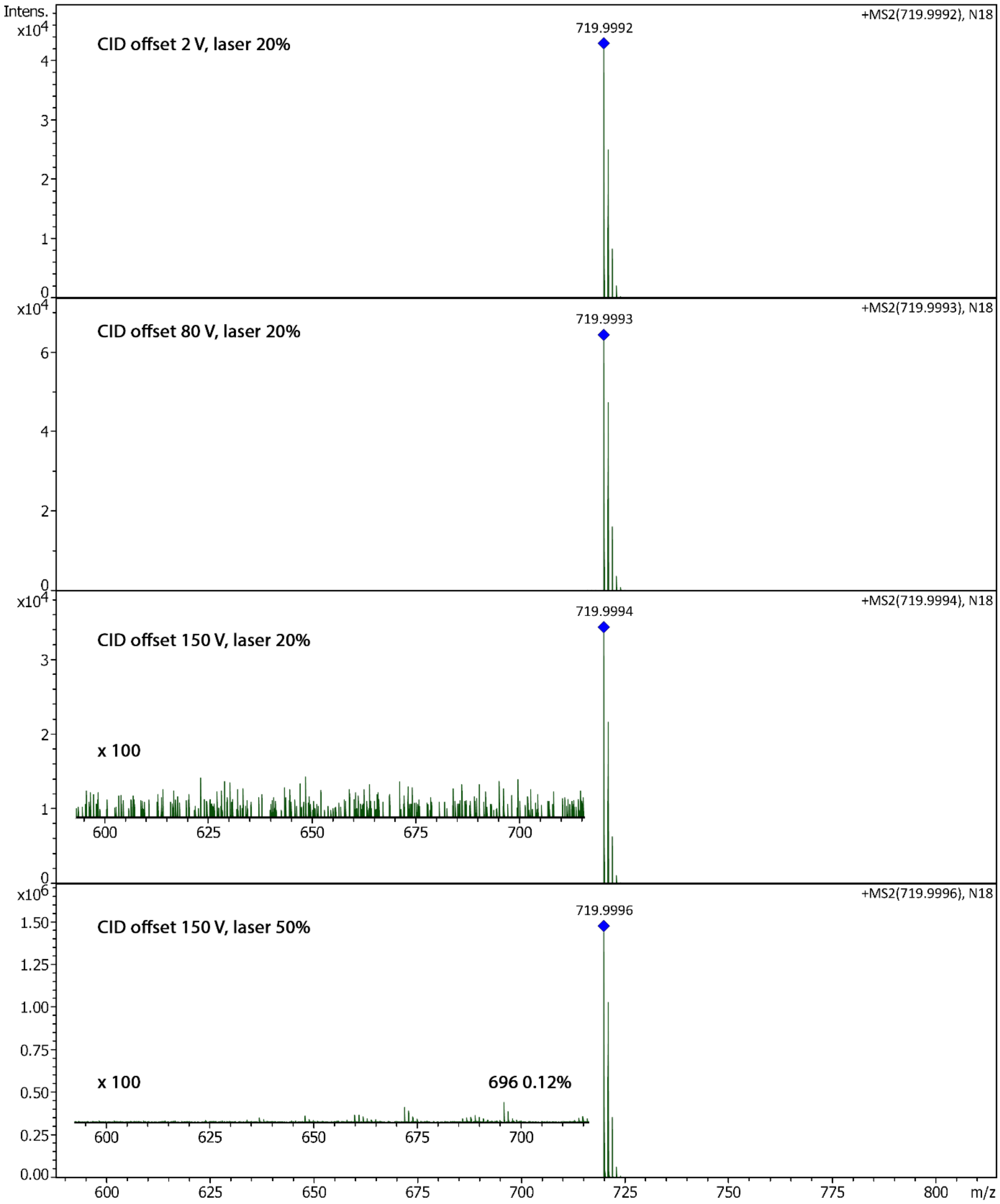

Fig. S3–S6. Measurements leading to the data provided in Table 1 of the manuscript covering $1/K_0$ = 0.95–1.45 (ramp time 500 ms, accumulation 10 ms, 2.80 mbar), and thus, the ions from $C_{56}^{+\bullet}$ to $C_{112}^{+\bullet}$. In **Fig. S3**, the calibration report taken from the first analysis shows that mass calibration and TIMS calibration were based on Tune Mix. The peaks at nominal *m/z* 622 ($1/K_0$ = 0.991), 922 ($1/K_0$ = 1.199), and 1222 ($1/K_0$ = 1.393) were within that range and provided CCS calibration for the subsequent LDI-TIMS runs. **Figs. S4 to S6** are screenshots of the three LDI-TIMS measurements while analyzed in DA 6.1. There, the base peak mobilogram (BPC) with compounds assigned is on top, then the spectrum shows the sum across the entire range, next three example compound spectra of fullerenes reveal the level of separation, and finally, the compound list provides CCS values or in some cases just $1/K_0$ values of the compounds. In these cases, the Compass Mobility Calculator was used to manually fill the gaps.

Calibration Report

Analysis Info

Analysis	D:\Projekte\LDI-TIMS_Fullerene_2024\Messungen\tims10493_0_G1_MS.d	Acquisition Date	05.06.2024 14:55:12
Method	MALDIpos_250-5000.m	Operator	TOF-User
Sample	tims10493	Instrument	timsTOF fleX 1859745.20462
Comment	Fullerene soot, LDI 40%, 5days old, TIMS custom 0.95-1.45m 500 ms, 10ms, 2.80 mbar		

Acquisition Parameter

Source Type	MALDI	Ion Polarity	Positive	Set Nebulizer	0.5 bar
Scan Begin	250 m/z	Set Capillary	3500 V	Set Dry Heater	200 °C
Scan End	5000 m/z	Set Multipole RF	480.0 Vpp	Set Dry Gas	3.0 l/min
		Set Collision Cell RF	2500.0 Vpp	ICC active	Off
		IMS Active	On	ICC Target	2000000 cts.
		IMS Collision Cell In	300.0 V		

Calibration Status

Instrument calibration

Date:	05.06.2024 14:26:58
Polarity:	Positive
Calibration spectrum:	<unknown>
Reference mass list:	Tuning Mix ES-TOF (ESI)
Calibration mode:	Linear
Standard deviation:	0.316 ppm

Reference m/z	Resulting m/z	Intensity	Error [ppm]
622.0290	622.0292	401440	0.373
922.0098	922.0100	535648	0.223
1221.9906	1221.9906	559587	-0.041
1521.9715	1521.9710	411021	-0.283
1821.9523	1821.9515	227327	-0.422
2121.9331	2121.9326	220509	-0.250
2421.9140	2421.9142	103771	0.069
2721.8948	2721.8960	48139	0.432

Instrument mobility calibration

Date:	05.06.2024 14:52:29
Polarity:	Positive
Calibration mobilogram:	<unknown>
Reference mass list:	Tuning Mix ES-TOF CCS compendium (ESI)-highmass
Standard deviation:	0.036%

Reference mobility	Resulting mobility	Intensity	Error [%]
0.991	0.992	33124402	0.008
1.199	1.198	48099956	-0.025
1.393	1.394	65800848	0.018

S4.

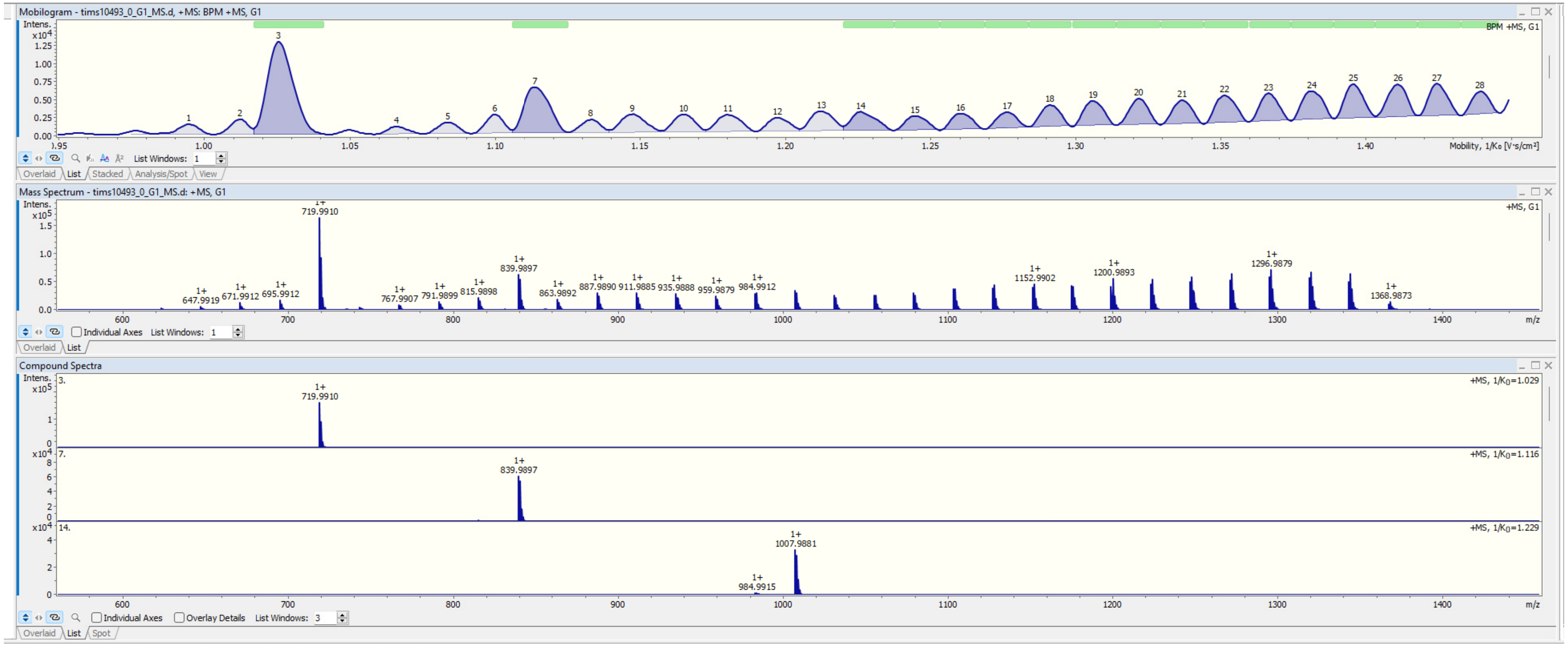

#	Int. Type	Max. m/z	Trace	Mobility 1/K0	I 1/K0	Resolution 1/K0	CCS [Å²]
1	Mobilogram	671.9912	BPM +MS, G1	0.995	1725	120.645	203.4
2	Mobilogram	695.9911	BPM +MS, G1	1.013	2395	141.648	206.8
3	Mobilogram	719.9910	BPM +MS, G1	1.026	13312	102.827	209.4
4	Mobilogram	767.9906	BPM +MS, G1	1.066	1414	133.708	217.4
5	Mobilogram	791.9899	BPM +MS, G1	1.084	2007	135.071	220.9
6	Mobilogram	815.9900	BPM +MS, G1	1.100	3084	152.232	224.1
7	Mobilogram	839.9897	BPM +MS, G1	1.114	6968	109.990	226.8
8	Mobilogram	863.9893	BPM +MS, G1	1.134	2373	144.204	230.6
9	Mobilogram	887.9890	BPM +MS, G1	1.148	3143	119.472	233.4
10	Mobilogram	911.9884	BPM +MS, G1	1.165	3136	135.792	236.9
11	Mobilogram	935.9888	BPM +MS, G1	1.181	3054	122.758	239.9
12	Mobilogram	959.9879	BPM +MS, G1	1.198	2670	137.810	243.3
13	Mobilogram	984.9912	BPM +MS, G1	1.213	3584	134.429	246.3
14	Mobilogram	1007.9881	BPM +MS, G1	1.226	3478	119.291	249.0
15	Mobilogram	1031.9874	BPM +MS, G1	1.245	2903	145.115	252.7
16	Mobilogram	1055.9876	BPM +MS, G1	1.261	3261	148.343	255.8
17	Mobilogram	1079.9878	BPM +MS, G1	1.277	3442	157.105	259.0
18	Mobilogram	1104.9903	BPM +MS, G1	1.292	4443	156.534	261.9
19	Mobilogram	1128.9899	BPM +MS, G1	1.307	5038	160.445	264.9
20	Mobilogram	1152.9902	BPM +MS, G1	1.322	5309	173.134	268.0
21	Mobilogram	1175.9860	BPM +MS, G1	1.337	5077	169.316	271.0
22	Mobilogram	1200.9892	BPM +MS, G1	1.352	5783	164.725	
23	Mobilogram	1224.9891	BPM +MS, G1	1.367	6069	183.012	276.8
24	Mobilogram	1248.9891	BPM +MS, G1	1.382	6419	167.558	279.8
25	Mobilogram	1272.9882	BPM +MS, G1	1.396	7334	185.535	

S5.

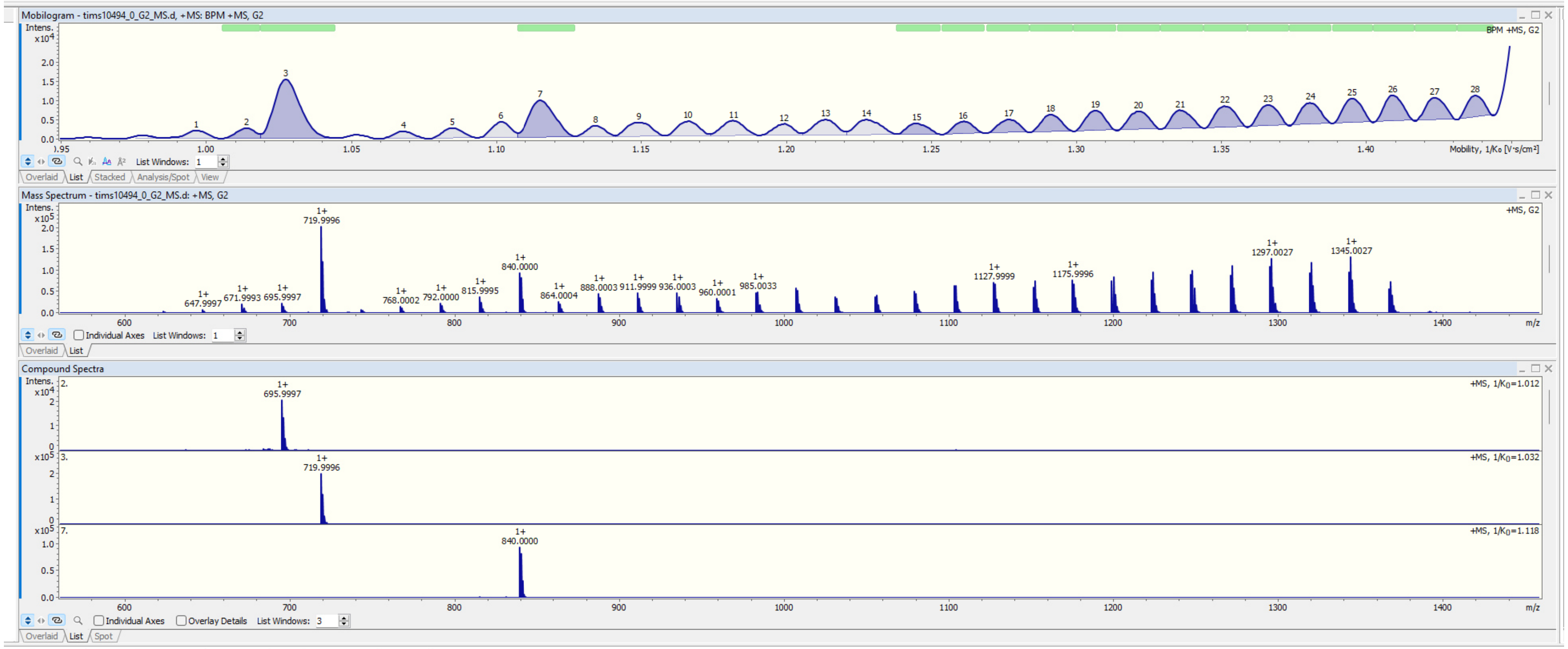

#	Int. Type	Max. m/z	Trace	Mobility 1/K0	I 1/K0	Resolution 1/K0	CCS [Å2]
1	Mobilogram	671.9993	BPM +MS, G2	0.997	2458	122.267	203.8
2	Mobilogram	695.9997	BPM +MS, G2	1.014	3089	126.206	207.2
3	Mobilogram	719.9996	BPM +MS, G2	1.028	15888	102.721	209.8
4	Mobilogram	768.0001	BPM +MS, G2	1.068	2241	134.680	217.8
5	Mobilogram	792.0000	BPM +MS, G2	1.085	3131	135.132	221.1
6	Mobilogram	815.9995	BPM +MS, G2	1.102	4726	141.827	224.4
7	Mobilogram	840.0000	BPM +MS, G2	1.116	10337	109.696	227.1
8	Mobilogram	864.0004	BPM +MS, G2	1.135	3686	149.876	230.9
9	Mobilogram	888.0003	BPM +MS, G2	1.150	4586	112.163	233.8
10	Mobilogram	911.9999	BPM +MS, G2	1.167	4870	131.335	237.2
11	Mobilogram	936.0003	BPM +MS, G2	1.182	5053	131.723	240.2
12	Mobilogram	960.0001	BPM +MS, G2	1.200	4191	148.760	243.7
13	Mobilogram	985.0034	BPM +MS, G2	1.214	5281	130.620	246.5
14	Mobilogram	1007.9999	BPM +MS, G2	1.228	5304	117.446	249.3
15	Mobilogram	1032.0006	BPM +MS, G2	1.245	4276	144.065	252.7
16	Mobilogram	1057.0036	BPM +MS, G2	1.261	4837	161.153	255.9
17	Mobilogram	1079.9998	BPM +MS, G2	1.277	5316	153.772	259.1
18	Mobilogram	1104.0000	BPM +MS, G2	1.292	6655	164.154	261.9
19	Mobilogram	1127.9999	BPM +MS, G2	1.307	7720	168.912	265.0
20	Mobilogram	1153.0035	BPM +MS, G2	1.322	7504	165.252	
21	Mobilogram	1175.9995	BPM +MS, G2	1.336	7780	164.792	270.8
22	Mobilogram	1201.0034	BPM +MS, G2	1.352	8848	167.608	273.8
23	Mobilogram	1225.0031	BPM +MS, G2	1.367	9155	171.354	
24	Mobilogram	1249.0032	BPM +MS, G2	1.381	9741	172.710	279.7
25	Mobilogram	1273.0031	BPM +MS, G2	1.396	10866	183.347	

S6.

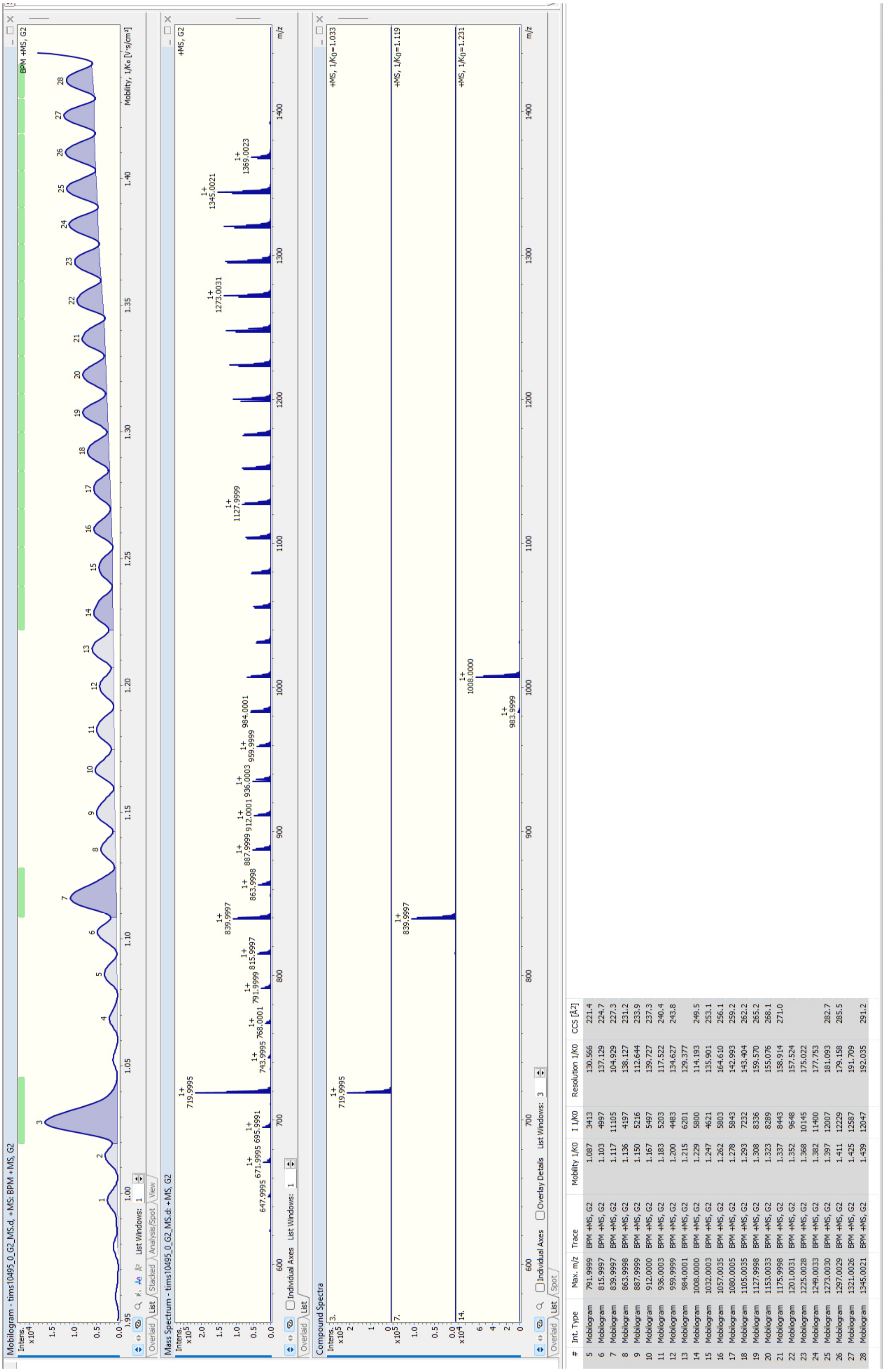

#	Int. Type	Max. m/z	Trace	Mobility 1/K0	I 1/K0	Resolution 1/K0	CCS [Å²]
5	Mobilogram	791.9999	BPM +MS, G2	1.087	3413	130.566	221.4
6	Mobilogram	815.9997	BPM +MS, G2	1.103	4997	137.129	224.7
7	Mobilogram	839.9997	BPM +MS, G2	1.117	11105	104.929	227.3
8	Mobilogram	863.9998	BPM +MS, G2	1.136	4197	138.127	231.2
9	Mobilogram	887.9999	BPM +MS, G2	1.150	5216	112.644	233.9
10	Mobilogram	912.0000	BPM +MS, G2	1.167	5497	139.727	237.3
11	Mobilogram	936.0003	BPM +MS, G2	1.183	5203	117.522	240.4
12	Mobilogram	959.9999	BPM +MS, G2	1.200	4483	134.627	243.8
13	Mobilogram	984.0001	BPM +MS, G2	1.215	6201	129.377	
14	Mobilogram	1008.0000	BPM +MS, G2	1.229	5800	114.193	249.5
15	Mobilogram	1032.0003	BPM +MS, G2	1.247	4621	135.901	253.1
16	Mobilogram	1057.0035	BPM +MS, G2	1.262	5803	164.610	256.1
17	Mobilogram	1080.0005	BPM +MS, G2	1.278	5843	142.993	259.2
18	Mobilogram	1105.0035	BPM +MS, G2	1.293	7232	143.404	262.2
19	Mobilogram	1127.9998	BPM +MS, G2	1.308	8336	159.570	265.2
20	Mobilogram	1153.0033	BPM +MS, G2	1.323	8289	155.076	268.1
21	Mobilogram	1175.9998	BPM +MS, G2	1.337	8443	158.914	271.0
22	Mobilogram	1201.0031	BPM +MS, G2	1.352	9648	157.524	
23	Mobilogram	1225.0028	BPM +MS, G2	1.368	10145	175.022	
24	Mobilogram	1249.0033	BPM +MS, G2	1.382	11400	177.753	
25	Mobilogram	1273.0030	BPM +MS, G2	1.397	12007	181.093	282.7
26	Mobilogram	1297.0029	BPM +MS, G2	1.411	12229	179.158	285.5
27	Mobilogram	1321.0026	BPM +MS, G2	1.425	12587	191.709	
28	Mobilogram	1345.0021	BPM +MS, G2	1.439	12047	192.035	291.2

Table S1. CCS value determinations of positive fullerene molecular ions

					TIMS 0.95-1.45, 500 ms, 10 ms, 2.60 mbar			TIMS 1.10-1.60, 500 ms, 10 ms, 2.30 mbar			TIMS 0.95-1.45, 500 ms, 10 ms, 2.80 mbar			TIMS 1.08-1.58, 500 ms, 10 ms, 2.80 mbar			TIMS 1.35-1.85, 500 ms, 10 ms, 2.65 mbar				TIMS 1.50-2.00, 500 ms, 10 ms, 2.45 mbar			TIMS 0.95-1.25 500ms 10ms 2.75 mbar		
				CCS [Å2]	experimental, May 31, 2024			experimental, May 31, 2024			experimental, June 4, 2024			experimental, June 4, 2024			experimental, June 4, 2024				experimental, June 4, 2024			experimental, June 14, 2024		
Entry No.	m/z (calc.)	Nominal m/z	Formula	Reference	10444	10446	10447	10440	10441	10442	10493	10494	10495	10496	10497	10498	10500	10501	10502	10503	10506	10507	10508	10696	10697	10698
1	671.9995	672	C56		202.6	202.2	202.0				203.4	203.8	204.0													
2	695.9995	696	C58		206.1	205.5	205.5				206.8	207.2	207.3													
3	719.9995	720	C60	210.0	208.8	208.2	208.1				209.4	209.8	209.9											209.2	209.6	209.5
4	743.9995	744	C62		213.6	213.0	212.8				214.2	214.6	214.8													
5	767.9995	768	C64		216.7	216.0	215.9				217.4	217.4	218.0													
6	791.9995	792	C66		220.0	219.4	219.3				220.9	221.1	221.4	221.2	221.3	221.3										
7	815.9995	816	C68		223.2	222.5	222.6				224.1	224.4	224.7	224.5	224.5	224.5										
8	839.9995	840	C70	226.6	226.0	225.2	225.0	225.4	225.3	225.5	226.8	227.1	227.3	227.1	227.3	227.3										
9	863.9995	864	C72		229.9	229.0	228.9	229.3	229.2	229.5	230.6	230.9	231.2	231.0	230.9	231.1										
10	887.9995	888	C74		232.6	231.7	231.6	232.0	232.1	232.3	233.4	233.8	233.9	233.8	233.9	233.8										
11	911.9995	912	C76	236.2	236.0	235.2	235.0	235.5	235.4	235.7	236.4	237.2	237.3	237.3	237.3	237.4										
12	935.9995	936	C78	239.2	239.0	238.3	238.0	238.4	238.3	238.5	239.9	240.2	240.4	240.3	240.3	240.4										
13	959.9995	960	C80		242.5	241.5	241.6	241.8	241.6	242.1	243.3	243.7	243.8	243.8	243.6	243.8										
14	983.9995	984	C82	245.2	245.3	244.4	244.4	244.8	244.7	244.8	246.3	246.5	246.7	246.7	246.5	246.7										
15	1007.9995	1008	C84	247.9	248.1	247.3	247.3	247.5	247.5	247.7	249.0	249.3	249.5	249.5	249.6	249.5										
16	1031.9995	1032	C86	251.1	251.6	250.8	250.5	251.1	251.0	251.3	252.7	252.7	253.1	252.1	252.9	253.0										
17	1055.9995	1056	C88	254.5	254.7	253.7	253.7	254.0	254.0	254.2	255.8	255.9	256.1	256.2	255.9	256.1										
18	1079.9995	1080	C90	257.3	257.6	256.8	256.7	257.1	257.0	257.2	259.0	259.1	259.2	259.2	259.0	259.2										
19	1103.9995	1104	C92	260.1	260.8	259.9	259.7	260.0	260.1	260.2	261.9	261.9	262.2	262.2	262.0	262.2										
20	1127.9995	1128	C94	263.3	263.5	262.7	262.7	263.0	263.0	263.0	264.9	265.0	265.2	265.0	265.0	265.2										
21	1151.9995	1152	C96	266.0	266.6	265.7	265.5	266.0	265.9	266.1	268.0	267.9	268.1	268.1	267.9	268.1										
22	1175.9995	1176	C98		269.6	268.7	268.4	268.9	268.9	269.2	271.0	270.8	271.0	271.0	271.0	271.1										
23	1199.9995	1200	C100		272.6	271.6	271.5	271.7	271.9	272.0	273.9	273.8	273.9	274.0	273.9	274.1	272.3	274.3	274.3	274.1						
24	1223.9995	1224	C102		275.2	274.6	274.4	274.8	274.7	275.0	276.8	276.9	277.0	277.0	276.9	277.1	277.5	277.5	277.1	277.1						
25	1247.9995	1248	C104		278.3	277.3	277.3	277.6	277.4	277.8	279.8	279.7	279.8	279.9	279.8	279.8	280.4	280.4	280.0	280.0						
26	1271.9995	1272	C106		281.1	280.2	280.0	280.6	280.5	280.7	282.6	282.6	282.7	282.7	282.6	282.8	283.3	283.3	282.2	282.8						
27	1295.9995	1296	C108		284.0	283.1	282.9	283.4	283.4	283.5	285.6	285.3	285.5	285.7	285.4	285.6	286.2	286.2	285.8	285.9						
28	1319.9995	1320	C110		286.7	285.7	285.7	286.4	286.2	286.3	288.4	288.2	288.4	288.5	288.2	288.4	289.0	289.0	288.8	288.7						
29	1343.9995	1344	C112		289.5	288.7	288.5	288.9	288.9	289.1	291.3	291.0	291.2	291.3	291.1	291.3	291.8	291.8	291.6	291.5						
30	1367.9995	1368	C114		292.3	291.4	291.2	291.9	291.8	291.9				294.1	292.9	294.0	294.7	294.7	294.4	294.3						
31	1391.9995	1392	C116					294.6	294.5	294.8				296.8	296.7	296.8	297.5	297.5	297.1	297.2						
32	1415.9995	1416	C118					297.3	297.3	297.4				299.5	299.3	299.5	300.2	300.2	299.9	299.9						
33	1439.9995	1440	C120					300.0	299.8	300.0				302.2	302.1	302.2	303.0	303.0	302.7	302.6						
34	1463.9995	1464	C122					302.7	302.7	302.8				305.0	304.8	304.9	305.7	305.7	305.3	305.3	306.3	305.4	305.5			
35	1487.9995	1488	C124					305.3	305.3	305.4				307.6	307.5	307.5	308.4	308.4	308.0	308.0	308.8	308.0	308.1			
36	1511.9995	1512	C126					308.0	308.0	307.9				310.3	310.1	310.2	311.0	311.0	310.7	310.7	311.6	310.7	310.8			
37	1535.9995	1536	C128					310.6	310.5	310.7				312.9	312.8	312.8	313.7	313.7	313.3	313.3	314.3	313.4	313.3			
38	1559.9995	1560	C130					313.2	313.1	313.2				315.6	315.4	315.4	316.4	316.4	316.0	316.0	316.9	316.0	316.0			
39	1583.9995	1584	C132					315.7	315.6	315.8				317.8	317.7	317.7	319.0	319.0	318.6	318.6	319.6	318.7	318.5			
40	1607.9995	1608	C134					318.3	318.3	318.5							321.6	321.6	321.2	321.2	322.1	321.3	321.2			
41	1631.9995	1632	C136					320.7	320.7	320.7							324.2	324.2	323.8	323.8	324.8	323.8	323.7			
42	1655.9995	1656	C138													326.7	326.7	326.4	326.4	327.3	326.4	326.1			
43	1679.9995	1680	C140													329.3	329.3	329.0	328.9	330.0	329.0	328.8			
44	1703.9995	1704	C142													331.9	331.9	331.5	331.5	332.5	331.6	331.2			
45	1727.9995	1728	C144													334.4	334.4	334.1	334.0	335.0	334.1	333.7			
46	1751.9995	1752	C146													336.9	336.9	336.6	336.6	337.5	336.5	336.3			
47	1775.9995	1776	C148													339.4	339.4	339.1	339.1	340.0	339.2	338.8			
48	1799.9995	1800	C150													341.9	341.9	341.5	341.5	342.6	341.6	341.2			
49	1823.9995	1824	C152													344.4	344.4	344.1	344.0	345.0	344.1	343.7			
50	1847.9995	1848	C154													347.0	347.0	346.5	346.5	347.5	346.6	346.1			
51	1871.9995	1872	C156													349.3	349.3	349.0	348.9	350.0	349.1	348.5			
52	1895.9995	1896	C158													351.8	351.8	351.4	351.4	352.4	351.5	350.9			
53	1919.9995	1920	C160													354.2	354.2	353.8	353.8	354.9	354.0	353.4			
54	1943.9995	1944	C162													356.6	356.6	356.3	356.3	357.3	356.4	355.7			
55	1967.9995	1968	C164													359.1	359.1	358.6	358.6	359.7	358.8	358.2			
56	1991.9995	1992	C166													361.4	361.4	361.1	361.0	362.2	361.2	360.5			
57	2015.9995	2016	C168													363.9	363.9	363.4	363.4	364.4	363.6	362.9			
58	2039.9995	2040	C170													366.2	366.2	365.8	365.7	367.0	366.0	365.3			
59	2063.9995	2064	C172													368.5	368.5	368.1	368.1	369.4	368.4	367.5			
60	2087.9995	2088	C174													370.7	370.7	370.1	370.4	371.7	370.8	369.9			
61	2111.9995	2112	C176																	374.1	373.2	372.3			
62	2135.9995	2136	C178																	376.4	375.5	374.5			
63	2159.9995	2160	C180																	378.8	377.8	376.8			
64	2183.9995	2184	C182																	381.2	380.1	379.1			
65	2207.9995	2208	C184																	383.4	382.5	381.3			
66	2231.9995	2232	C186																	385.8	384.8	383.6			
67	2255.9995	2256	C188																	388.1	387.0	385.8			
68	2279.9995	2280	C190																	390.3	389.3	388.2			
69	2303.9995	2304	C192																	392.6	391.5	390.4			
70	2327.9995	2328	C194																	394.8	393.8	392.5			
71	2351.9995	2352	C196																	397.2	396.0	394.7			
72	2375.9995	2376	C198																	399.3	398.2	396.8			
73	2399.9995	2400	C200																	401.3		398.9			
74	2423.9995	2424	C202																			400.9			
75	2447.9995	2448	C204																						
76	2471.9995	2472	C206																						
77	2495.9995	2496	C208																						
78	2519.9995	2520	C210																						
79	2543.9995	2544	C212																						
80	2567.9995	2568	C214																						
81	2591.9995	2592	C216																						
82	2615.9995	2616	C218																						
83	2639.9995	2640	C220																						
84	2663.9995	2664	C222																						
85	2687.9995	2688	C224																						
86	2711.9995	2712	C226																						
87	2735.9995	2736	C228																						
88	2759.9995	2760	C230																						
89	2783.9995	2784	C232																						
90	2807.9995	2808	C234																						
91	2831.9995	2832	C236																						
92	2855.9995	2856	C238																						
93	2879.9995	2880	C240																						

	35%, TIMS 0.95-1.25 500ms 10ms 2.75 mbar			35%, TIMS 1.10-1.25 500ms 10ms 2.75 mbar			TIMS 1.60-2.10, 500 ms, 10 ms, 2.25 mbar				TIMS 1.85-2.25 500 ms, 10 ms, 2.30 mbar				TIMS 1.70-2.25, 500 ms, 10 ms, 2.40 mbar			TIMS 1.85-2.25, 500 ms, 10 ms, 2.40 mbar			TIMS 1.70-2.10, 500 ms, 10 ms, 2.40 mbar						
	experimental, June 14, 2024			experimental, June 14, 2024			experimental, June 14, 2024				experimental, June 21, 2024				experimental, July 29, 2024			experimental, July 29, 2024			experimental, July 29, 2024			CCS [Å2]			
Entry No.	10699	10700	10701	10703	10704	10705	10716	10717	10718	10719	10832	10833	10834	10835	11516	11517	11518	11520	11521	11522	11524	11525	11526	Average	Stand. Dev. [%]	Delta CCS to Ref. [Å2]	Delta CCS to Ref. [%]
1	203.6	203.5	203.4																					203.2	0.33		
2	207.0	207.0	206.9																					206.6	0.32		
3	209.5	209.6	209.4																					209.3	0.27	0.8	0.4
4	214.3	214.2	214.2																					214.0	0.30		
5	217.3	217.1	217.1																					217.0	0.30		
6	220.8	220.8	220.6																					220.7	0.32		
7	223.9	224.1	223.8	224.4																				223.9	0.31		
8	226.6	226.7	226.6	227.2	227.0	226.7																		226.5	0.35	0.2	0.1
9	230.3	230.4	203.3	231.0	230.8	230.5																		228.8	2.72		
10	233.3	233.3	233.2	233.9	233.6	233.3																		233.1	0.34		
11	236.6	236.6	236.4	237.2	237.0	237.0																		236.5	0.33	-0.3	-0.1
12	239.6	239.8	239.4	240.0	240.0	239.8																		239.5	0.34	-0.3	-0.1
13	242.9	243.2	242.9	243.7	243.6	243.2																		242.9	0.34		
14	245.7	245.9	245.7	246.7	246.5	246.2																		245.8	0.34	-0.6	-0.2
15	248.7	248.8	248.7	249.3	249.2	248.7																		248.6	0.33	-0.7	-0.3
16			252.2			252.4																		252.0	0.34	-0.9	-0.3
17																								255.0	0.40	-0.5	-0.2
18																								258.1	0.41	-0.8	-0.3
19																								261.1	0.39	-1.0	-0.4
20																								264.0	0.40	-0.7	-0.3
21																								267.0	0.40	-1.0	-0.4
22																								270.0	0.39		
23																								273.1	0.39		
24																								276.2	0.41		
25																								279.1	0.42		
26																								281.9	0.40		
27																								284.8	0.41		
28																								287.7	0.42		
29																								290.5	0.42		
30																								293.0	0.44		
31																								296.4	0.39		
32																								299.1	0.39		
33																								301.8	0.41		
34																								304.8	0.39		
35																								307.4	0.39		
36																								310.1	0.39		
37																								312.7	0.39		
38																								315.4	0.40		
39																								317.9	0.41		
40																								320.5	0.45		
41							324.7	323.7	324.0	324.3														323.0	0.48		
42							327.3	327.4	326.7	326.8														326.6	0.11		
43							329.8	330.0	329.3	329.5														329.2	0.11		
44							332.3	332.5	331.8	331.9														331.7	0.12		
45							334.9	335.1	334.8	334.5														334.2	0.11		
46							337.4	337.6	336.8	337.0														336.8	0.11		
47							339.9	340.0	339.4	339.5														339.3	0.10		
48							342.3	342.4	341.9	342.0														341.7	0.12		
49							344.7	344.9	344.4	344.4					344.3	345.1	344.2				344.3	344.7	344.3	344.2	0.11		
50							347.3	347.4	346.8	346.9					346.9	347.4	346.7				346.8	347.2	346.8	346.7	0.12		
51							349.6	349.8	349.3	349.3					349.3	349.9	349.0				349.2	349.6	349.2	349.2	0.12		
52							252.0	252.2	351.7	351.8					351.9	352.4	351.5				351.6	351.9	351.5	351.6	0.12		
53							354.5	354.7	354.1	354.2					354.2	354.7	353.9				354.0	354.3	354.0	354.0	0.12		
54							356.9	357.1	356.6	356.7					356.6	357.0	356.3				356.4	356.7	356.4	356.5	0.12		
55							359.3	359.5	358.9	358.9					359.0	359.4	358.8				358.9	359.1	358.7	358.9	0.12		
56							361.7	361.9	361.4	361.5					361.4	361.8	360.9				361.3	361.4	361.1	361.3	0.13		
57							364.0	364.2	363.8	363.9					363.8	364.1	363.4				363.5	363.8	363.5	363.6	0.12		
58							366.4	366.5	366.2	366.2					366.1	366.4	365.9				365.9	366.2	365.8	366.0	0.13		
59							368.8	368.9	368.6	368.6					368.5	368.6	368.2				368.3	368.5	368.2	368.4	0.15		
60							371.1	371.2	370.8	371.0					370.9	371.1	370.5				370.5	370.8	370.5	370.6	0.15		
61							373.5	373.6	373.3	373.3					373.3	373.4	372.9				372.9	373.1	372.7	373.2	0.11		
62							375.7	375.9	375.5	375.5	375.5	375.2	375.3	375.4	375.4	375.7	375.2	375.1	375.4	375.4	375.3	375.5	375.0	375.4	0.10		
63							378.0	378.2	377.9	377.9	377.7	377.6	377.7	377.7	377.9	377.9	377.7	377.5	377.5	377.7	377.5	377.7	377.4	377.7	0.10		
64							380.3	380.5	380.3	380.3	380.0	379.9	380.0	380.0	380.1	380.3	379.8	379.8	380.1	380.0	379.8	380.0	379.7	380.1	0.10		
65							382.4	382.7	382.6	382.6	382.4	382.2	382.4	382.2	382.3	382.4	382.2	382.1	382.2	382.3	382.1	382.3	381.9	382.3	0.10		
66							384.8	385.0	384.9	384.9	384.6	384.5	384.5	384.5	384.7	384.7	384.5	384.3	384.5	384.4	384.4	384.6	384.1	384.6	0.11		
67							387.2	387.4	387.2	387.2	386.9	386.7	386.8	386.8	387.0	387.1	386.7	386.5	386.7	386.8	386.6	386.8	386.4	386.9	0.11		
68							389.3	389.6	389.4	389.4	389.2	389.0	388.9	389.2	389.3	389.2	389.0	388.9	389.0	389.1	388.9	389.2	388.6	389.2	0.10		
69							391.6	391.9	391.7	391.7	391.4	391.3	391.4	391.3	391.5	391.5	391.2	391.1	391.2	391.4	391.2	391.3	390.8	391.4	0.11		
70							393.9	394.1	393.9	394.0	393.6	393.6	393.6	393.6	393.7	393.6	393.6	393.3	393.5	393.7	393.3	393.6	393.1	393.6	0.11		
71							396.0	396.3	396.3	396.1	395.9	395.8	395.9	395.8	396.1	395.9	395.6	396.6	395.7	395.9	395.6	395.7	395.3	395.9	0.12		
72							398.3	398.6	398.5	398.5	398.1	398.0	398.1	398.0	398.2	398.1	398.0	397.8	397.9	398.0	397.8	397.9	397.5	398.1	0.12		
73							400.5	400.8	400.7	400.6	400.3	400.2	400.3	400.3	400.3	400.0	400.3	400.1	400.1	400.2	400.0	400.2	399.6	400.2	0.12		
74							402.8	402.9	402.8	402.8	402.6	402.4	402.3	402.5	402.5	402.4	402.5	402.2	402.3	402.6	402.1	402.3	401.8	402.4	0.11		
75							404.9	405.2	405.1	404.9	404.7	404.7	404.7	404.6	404.8	404.5	404.6	404.4	404.3	404.6	404.4	404.5	404.0	404.6	0.07		
76							407.1	407.4	407.3	407.2	407.0	406.9	406.9	406.9	406.9	406.6	406.9	406.6	406.7	406.9	406.5	406.7	406.1	406.9	0.07		
77							409.2	409.5	409.6	409.5	409.2	409.0	409.1	409.0	409.2	408.9	408.9	408.7	408.8					409.1	0.07		
78							411.4	411.5	411.7	411.6	411.4	411.3	411.3	411.2	411.4	411.1	411.3	410.9	411.0		410.8	411.0	410.5	411.2	0.07		
79							413.6	413.8	413.8	413.8	413.5	413.4	413.4	413.3	413.4	413.3	413.5	412.0	413.2	413.2	412.9	413.1	412.6	413.3	0.11		
80							415.8	415.8	416.0	416.1	415.6	415.6	415.6	415.6	415.7			415.2	415.1	415.4	414.9	415.3	414.8	415.5	0.09		
81							417.9	418.0	417.9	418.0	417.7	417.6	417.7	417.6	417.8	417.4	417.7	417.3	417.4			417.3		417.7	0.06		
82								420.2		419.9	419.9	419.9	419.8	419.9	419.9	421.6	419.7	419.4	419.3	419.8			418.7	419.8	0.15		
83											422.0	422.0	422.0	422.0	421.9	423.5	422.0	421.5	421.6	421.8				422.0	0.12		
84											424.2	424.2	423.9	424.1	424.3	425.7	428.4	423.8	423.6	423.9				424.6	0.32		
85											426.3	426.1	426.2	426.2	428.2			425.8	425.7	426.2				426.3	0.17		
86											428.3	428.3	428.3	428.4	430.5	429.9		427.7	427.8	428.0				428.6	0.21		
87											430.5	430.5	430.3	430.5	432.6			430.1	429.9	430.1				430.6	0.19		
88											432.6	432.5	432.4	432.5	436.7			431.8		432.3				433.0	0.36		
89											434.7	434.6	434.7	434.7				434.1	434.0	434.3				434.4	0.06		
90											436.8	436.7	436.7	436.6				436.2		436.6				436.6	0.04		
91											438.9	438.7	438.7	438.9						440.6				439.2	0.17		
92											441.0	440.9	440.8	440.9						442.9				441.3	0.18		
93											443.0	442.9	442.8	442.9										442.9	0.02		

Legend to descriptions of data sets

TIMS 0.95-1.45, 500 ms, 10 ms, 2.80 mbar			This line provides basic TIMS setting used for the data set
experimental, June 4, 2024			Date when data set was acquired
10493	10494	10495	File numbers of each measurement

Reference	Data taken from Weis, P.; Hennrich, F.; Fischer, R.; Schneider, E. K.;
	Neumaier, M.; Kappes, M. M. Probing the structure of giant
	fullerenes by high resolution trapped ion mobility spectrometry.
210.0	Phys. Chem. Chem. Phys. 2019, 21 (35), 18877-18892.

280.0	Black CCS values were automatically
282.9	delivered by DataAnalysis 6.1
285.7	Blue CCS values were manually added by using the
288.5	1/Ko value and Compass Mobility Calculator Utility
291.2	

Table S2.

CCS Determinations based on Fullerenes					TIMS 0.95-1.45, 500 ms, 10 ms, 2.80 mbar			TIMS 1.08-1.58, 500 ms, 10 ms, 2.80 mbar			TIMS 1.10-1.60, 500 ms, 10 ms, 2.75 mbar			TIMS 1.35-1.85, 500 ms, 10 ms, 2.60 mbar				TIMS 1.65-2.15, 500 ms, 10 ms, 2.35 mbar								
				CCS [Å2]	experimental, June 6, 2024			experimental, June 6, 2024			experimental, June 14, 2024			experimental, Aug 2, 2024				experimental, Aug 2, 2024					CCS [Å2]			
Compound	m/z (calc.)	Nominal m/z	Formula	Reference							10706	10708	10709	11595	11596	11597	11598	11600	11601	11602	11603	11604	Average	Stand. Dev. [%]	Delta CCS [Å2]	Delta CCS [%]
TuneMix	622.0290	622	C12H19F12N3O6P3	**202.96**	202.4	202.8	202.7																202.6	0.1	0.3	0.2
TuneMix	922.0098	922	C18H19F24N3O6P3	**243.20**	242.6	242.9	242.9	242.6	242.7	242.7	242.2	242.7	242.7										242.7	0.1	0.5	0.2
TuneMix	1221.9906	1222	C24H19F36N3O6P3	**282.20**	280.8	280.8	280.9	281.6	281.7	281.7	281.2	281.5	281.5	281.1	281.2	281.1	281.5						281.3	0.1	0.9	0.3
TuneMix	1521.9715	1522	C30H19F48N3O6P3	**316.96**				316.7	316.6	316.7	316.5	316.6	316.6	317.3	317.1	317.0	317.2						316.8	0.1	0.1	0.0
TuneMix	1821.9523	1822	C36H19F60N3O6P3	**351.25**										352.5	351.8	351.8	351.7	352.2	351.8	351.3	351.4	351.4	351.8	0.1	-0.5	-0.1
TuneMix	2121.9332	2122	C42H19F72N3O6P3	**383.03**														384.3	383.8	383.2	383.5	383.5	383.7	0.1	-0.6	-0.2
TuneMix	2421.9140	2422	C48H19F84N3O6P3	**412.96**														414.4	413.7	413.2	413.6	413.6	413.7	0.1	-0.7	-0.2
TuneMix	2721.8948	2722	C54H19F96N3O6P3	**441.21**																						

CCS Determinations based on Fullerenes					TIMS 1.40-1.90, 500 ms, 10 ms, 2.50 mbar			TIMS 1.40-1.90, 500 ms, 10 ms		TIMS 1.35-1.85, 500 ms, 10 ms, 2.45 mbar				TIMS 0.95-1.45, 500 ms, 10 ms, 2.69 mbar			TIMS 1.35-1.85, 500 ms, 10 ms, 2.71 mbar		
					experimental, June 6, 2024			experimental, June 6, 2024		experimental, Aug 2, 2024				experimental, July 12, 2024			experimental, July 18, 2024		
			[M+H]+ Ion					10531	10532	11605	11607	11608	11609	11295	11296	11297	11439	11440	11441
Leucine-Enkephalin	556.2766	556	C28H38N5O7	**229.80**										225.5	225.6	225.8			
Angiotensin II	1046.5418	1046	C50H72N13O12	**313.87**	312.7	312.0	312.0			313.1	313.6	313.6	313.6				312.9	312.9	312.9
Angiotensin I	1296.6848	1297	C62H90N17O14	**356.14**	353.5	353.3	353.0	354.9	353.8	355.1	355.3	355.4	355.2				355.2	355.2	355.1
Substance P	1347.7354	1348	C63H99N18O13S	**360.54**	359.7	359.6	359.1	360.9	359.9	361.3	361.5	361.6	361.4				361.5	361.2	361.2
				**stepped field**															

CCS Determinations based on Fullerenes (continued)	TIMS 0.95-1.45, 500 ms, 10 ms, 2.69 mbar			TIMS 0.95-1.35, 500 ms, 10 ms, 2.75 mbar			TIMS 0.90-1.30, 500 ms, 10 ms, 2.70 mbar					TIMS 1.40-1.90, 500 ms, 10 ms, 2.55 mbar				TIMS 1.70-1.85, 500 ms, 10 ms, 2.55 mbar							
	experimental, July 18, 2024			experimental, June 7, 2024			experimental, June 21, 2024					experimental, June 21, 2024				experimental, June 21, 2024							
	11435	11436	11437	10575	10576	10578	10806	10808	10809	10811	10812	10814	10815	10817	10818	10820	10821	10823	10824	Average	Stand. Dev. [%]	Delta CCS [Å2]	Delta CCS [%]
Leucine-Enkephalin	225.1	226.1	225.7	227.2	227.2	226.7	225.9	226.6	226.6	226.7	226.6									226.1	0.3	3.7	1.6
Angiotensin II												313.8	314.0	313.1	313.3					313.1	0.2	0.8	0.2
Angiotensin I												355.3	355.5	353.9	353.8	353.5	353.5	353.8	353.8	354.4	0.2	1.7	0.5
Substance P												361.1	361.7	360.0	360.1	359.6	359.6	359.9	359.9	360.5	0.2	0.0	0.0

CCS Determinations based on TuneMix as Control					TIMS 1.35-1.85, 500 ms, 10 ms, 2.70 mbar			TIMS 0.95-1.45-1.90, 500 ms, 10 ms, 2.70 mbar						
					experimental, July 12, 2024			experimental, July 12, 2024						
			[M+H]+ Ion		11285	11286	11287	11289	11290	11291				
Leucine-Enkephalin	556.2766	556	C28H38N5O7	**229.80**				227.1	227.2	227.0	227.1	0.0	2.7	1.2
Angiotensin II	1046.5418	1046	C50H72N13O12	**313.87**	313.8	313.6	313.5				313.6	0.0	0.2	0.1
Angiotensin I	1296.6848	1297	C62H90N17O14	**356.14**	355.3	354.7	354.5				354.8	0.1	1.3	0.4
Substance P	1347.7354	1348	C63H99N18O13S	**360.54**	361.4	360.9	360.8				361.0	0.1	-0.5	-0.1
